# AbsIDconvert: An absolute approach for converting genetic identifiers at different granularities

**DOI:** 10.1186/1471-2105-13-229

**Published:** 2012-09-12

**Authors:** Fahim Mohammad, Robert M Flight, Benjamin J Harrison, Jeffrey C Petruska, Eric C Rouchka

**Affiliations:** 1 Department of Computer Engineering and Computer Science, University of Louisville, Louisville, KY, 40292, USA; 2 Department of Anatomical Sciences & Neurobiology, School of Medicine, University of Louisville, Louisville, KY, 40292, USA; 3 Kentucky Spinal Cord Injury Research Center, Department of Neurological Surgery, University of Louisville, Louisville, KY, 40292, USA; 4 Department of Pathology, Beth Israel Deaconess Medical Center, Harvard Medical School, Boston, MA, 02215, USA

**Keywords:** Annotation, Gene ID conversion, Meta-analysis, Genomic range, Interval trees, Comparative analysis, Granularity, Universal identifier, AbsIDconvert

## Abstract

**Background:**

High-throughput molecular biology techniques yield vast amounts of data, often by detecting small portions of ribonucleotides corresponding to specific identifiers. Existing bioinformatic methodologies categorize and compare these elements using inferred descriptive annotation given this sequence information irrespective of the fact that it may not be representative of the identifier as a whole.

**Results:**

All annotations, no matter the granularity, can be aligned to genomic sequences and therefore annotated by genomic intervals. We have developed *AbsIDconvert*, a methodology for converting between genomic identifiers by first mapping them onto a common universal coordinate system using an interval tree which is subsequently queried for overlapping identifiers. *AbsIDconvert* has many potential uses, including gene identifier conversion, identification of features within a genomic region, and cross-species comparisons. The utility is demonstrated in three case studies: 1) comparative genomic study mapping plasmodium gene sequences to corresponding human and mosquito transcriptional regions; 2) cross-species study of Incyte clone sequences; and 3) analysis of human Ensembl transcripts mapped by Affymetrix^®;^ and Agilent microarray probes. *AbsIDconvert* currently supports ID conversion of 53 species for a given list of input identifiers, genomic sequence, or genome intervals.

**Conclusion:**

*AbsIDconvert* provides an efficient and reliable mechanism for conversion between identifier domains of interest. The flexibility of this tool allows for custom definition identifier domains contingent upon the availability and determination of a genomic mapping interval. As the genomes and the sequences for genetic elements are further refined, this tool will become increasingly useful and accurate. *AbsIDconvert* is freely available as a web application or downloadable as a virtual machine at: http://bioinformatics.louisville.edu/abid/.

## Background

The Nucleic Acid Research (NAR) 2012 database issue [1] features 1,380 databases covering various aspects of molecular biology including sequences, gene expression, structures, pathways and diseases. Most of these databases are independent of each other and have been created as a result of the respective developers’ domain of interest and resource limitations. Due to a lack of standard naming conventions, most of these databases prefer to assign their own custom generated identifiers (IDs) to the biological entities. Major public databases such as GenBank [2] and RefSeq [3] use accession numbers, Gene Ontology (GO) [4] uses a naming convention from organism specific databases, the HUGO (Human Genome Organization) Gene Nomenclature Committee (HGNC) [5] uses the gene symbol and a custom generated ID, Entrez [6] uses numeric integers, sequencing projects use systematic names and biologists sometimes use additional aliases. As an example, the breast cancer early onset gene has the official gene symbol of BRCA2 provided by HGNC and an associated ID 1101, Ensembl [7] gene ID ENSG00000139618, OMIM (Online Mendelian Inheritance in Man) [8] ID 600185, HPR (Human Protein Reference) database [9,10] ID 02554, RefSeq ID NM_000059, GenBank Accession U43746, Entrez Gene ID 675, VEGA (the Vertebrate Genome Annotation database) [11] gene ID OTTHUMG00000017411, UCSC [12,13] gene ID uc001uub.1, UniProt [14] ID P51587, and gene aliases FAD, FAD1, BRCC2, FANCD1, FACD, FANCD.

Fortunately, there is a wealth of information available to the research community in a wide variety of databases. However, it is often difficult to extract or integrate information about a particular biological entity from multiple resources. For instance, a researcher may be interested in extracting functional information spread across different databases for a biological entity such as a gene or a protein; comparing two independent pathways which use different types of identifiers; or comparing results across species, platforms or labs. The lack of a common identifier across these heterogeneous and sometimes redundant biological databases makes the functional analysis of biological data tedious, time consuming, and error prone.

One solution to handle heterogeneous databases is to use a global identifier for annotations such as the one described by MIRIAM (Minimum Information Requested In the Annotation of biochemical Model) [15]. MIRIAM requires a global identifier to contain both the data source as well as an internal identifier. For example, *urn:miriam:hgnc:brca2* is composed of *urn:miriam* that defines the notation to be a URN (Uniform Resource Name) using the MIRIAM scheme with data type *hgnc* and identifier *brca2*. This method appears promising and has the potential to solve some of the previously mentioned problems, but very few databases follow this standard. Another solution is to manually search for these genes one by one in publicly available databases such as Entrez, KEGG [16,17], or GEO [18,19] and infer their functionality. This method is fruitful when the number of genes is small, but is impractical for high throughput experiments, where the number of gene fragments can be on the order of tens of thousands or more. A third solution is to use an ID converter tool that uses a database to store all possible annotations where a list of IDs may be input as a query which is then converted into the corresponding target IDs in a precise and efficient way.

**Figure 1:**
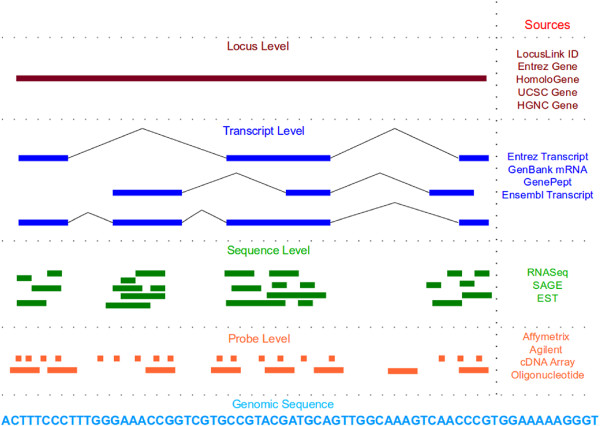
Granularity of annotations.

One difficulty in the development and maintenance of such conversion tools is the varying granularity of the identifiers. More specifically, the data generated by biological experiments may be at the locus, transcript, sequence or probe level, with varying coverage of a region of interest (Figure 1). This granularity ranges from very fine, at the level of DNA microarrays (tens of bases in length, containing probe level information relevant to only a short region of the corresponding mRNA molecule) through coarser granularity with sequence reads (few hundreds), transcripts (thousands), loci, and chromosomes. It is also possible that annotations at the same level may have different granularities. For example, among DNA microarray probes, Affymetrix^®;^ probes are usually short (25 bases) whereas Agilent probes are longer (60 bases) and cDNA probes are generally ≥ 500 nucleotides in length. The relationships between entities at the same or different granularities may be either 1-1, 1-n, n-1 or n-m: for example, an Affymetrix^®;^ probe may span more than one EST; more than one such probe may be contained inside an EST; a cDNA probe may contain zero, one or more Affymetrix^®;^ and Agilent probes.

Another difficulty in the development of such tools is the dynamic nature of annotations. Of late, rapid advances in sequencing and their declining costs have enabled researchers to perform novel sequencing as well as resequencing projects. These result in an increased depth of coverage of a genomic sequence, with gaps being filled and repeats more accurately mapped. Sometimes, the sequence underlying a genetic entity may change, and on a less frequent basis the whole genomic sequence needs to be updated (as of August 1^*st*^, 2012, the currently available genome versions for human, mouse and rat are 19 (GRCh37), 10 (GRCm38) and 5 (RGSC 5.0) respectively). These changes may modify the structural and functional annotations of a genetic entity (GenBank, RefSeq and Ensembl are updated everyday). Frequent updates in annotations also create problems in the manufacturing of DNA microarrays. Microarray chips are designed and their probes are annotated using the current build of a specific genome. Regardless of the care taken in this design, the system will include flaws due to the combination of the delay inherent in the process of microarray design-manufacture-deployment (compounded by the latency to use) and the dynamic nature of annotations. Attempts to address these problems have been the focus of a number of previous studies. Gautlier et al. [20] found redundancies in the annotations of Affymetrix^®;^ probes at a sequence level that map to multiple RefSeq genes. Such ambiguities may result in inaccurate interpretations. AffyProbeMiner [21] uses RefSeq and GenBank’s validated complete coding sequences to regroup the probes on an Affymetrix^®;^ chip into consistent probe sets. In their study, regrouping of the probes affected almost 65% of the probes on the HG-U133A chip. Harbig et al. [22] reidentified the Affymetrix^®;^ U133 plus 2.0 GeneChip^®;^ array probes in an attempt to increase the reproducibility of microarray experiments. They used BLAST [23] to remap the probes against the genome and redefined approximately 37% of the probes. These studies suggest that redefinition or reorganization of probesets will improve the analytical accuracy of the microarray data, a process that would be greatly facilitated by a means for high-throughput query and mapping/comparison of given sequences (such as microarray probes) to other genomic annotations stored across a wide variety of databases.

## Currently available ID conversion tools

The problem of ID conversion persists even though a number of tools exist to address this problem. Some of these are generic and perform ID conversion for probes, genes, proteins, and additional annotations while others are more specific to DNA microarray probes. Organism support varies with many of the tools catering to either a single organism or a small set of comparable species. In addition, cross–species comparison is variable, with most methodologies providing only intra–species conversion. Almost every approach uses some sort of relational database with the unique identifier being Ensembl IDs, RefSeq IDs, or custom generated IDs. A brief description of some popular tools follows.

*DAVID* (Database for Annotation, Visualization and Integrated Discovery) [24-26] is a web based structural and functional annotation tool to extract biological meaning from a gene list. It uniquely generates custom IDs for querying a set of relations and is dependent on annotations from other databases. A component of *DAVID*, *DICT*[27] (DAVID gene ID Conversion Tool), facilitates ID conversion. *EASE*[28], developed by the DAVID Bioinformatics team, is a customizable, standalone, Windows^®;^ desktop software application, having similar analytic capabilities as that of *DAVID*. *Babelomics*[29,30] is an integrated web based tool for structural and functional annotation with an *ID converter* being one of its components. This component uses a universal index linked to Ensembl to create a database of 11 species. *g:Convert*[31], a component of *g:Profiler*, allows arbitrary conversion of genes, proteins and probes into one another. Every alias in *g:Profiler* is mapped through a three-level index of gene, transcript and protein Ensembl IDs. For each index level, all corresponding IDs are stored in the database. The *Hyperlink Management System and ID Converter System*[32] automatically updates and maintains hyperlink information among major public biological and chemical databases. It downloads data everyday from authoritative databases and produces a large correspondence table which is used to show the most up-to-date URL for genes of interest. Users can use CGI programs to create hyperlinks to this data. *Synergizer*[33] assigns a unique internally generated identifier, “peg”, to all external IDs that refer to the same biological entity. It mostly uses the NCBI “gene2accession” file to maintain a database of synonym relationships and produce a simple web interface. *MADGene*[34] uses correspondence tables and allows conversions in an efficient way. The *Clone/Gene ID Converter*[35], *MatchMiner*[36], the *Gene name converter* in *GeneMerge*[37], *RESOURCERER*[38] and *GeneLynx*[39] are additional ID conversion tools.

Some of the ID conversion tools are more specific, such as those that work only at the probe level. *GATExplorer*[40] is a web based tool for analysis and visualization of Affymetrix^®;^ probes at the genomic and transcriptomic level. It performs de–novo mapping of all the probes of Affymetrix^®;^’s expression and exon arrays against the transcriptome of the corresponding organism using BLAST and records the coordinates on the genome. Unmapped probes are mapped to an ncRNA database downloaded from RNAdb. Only the perfect match alignment is selected while mapping these probes. The location of a gene or probe on the genome can be visualized along with all the transcripts present in that region. *NetAffx*^™^[41], provided by Affymetrix^®;^, performs ID conversion of Affymetrix^®;^ probes for different organisms and has a feature to perform structural and functional annotation. *PLANdbAffy*[42] is a Probe-Level ANnotation database for Affymetrix^®;^ microarrays (HG-U133A, HG-U133B, HG-U133 plus 2.0, Human Exon 1.0, Human Gene 1.0) that uses BLAT [43] to map individual probes onto the human genome. These probes are then annotated using information extracted from RefSeq. *ProbeMatchDB*[44] uses a number of public databases to perform cross-species and cross-platform probe mapping. The database conversions are enabled by UniGene and HomoloGene identifiers. UniProt’s [45,46]*ID mapping tool* works on the gene and protein level and converts gene IDs into UniProt IDs and vice versa.

Some software tools have unique methods for mapping between different IDs. *Onto–Translate*[47,48] converts one type of IDs into another by calculating the optimal path between IDs, taking into account the “trustworthiness” of data contained in various databases. The *AliasServer*[49] uses a custom generated unique 64-bit reference identifier which is computed from the amino acid sequence using the CRC (Cyclic Redundancy Check) algorithm where each ID is a unique combination of species identifier, type of database and the ID itself.

Some databases/tools aid in ID conversion but do not function as a full fledged ID conversion tool. *BioMart*[50,51], earlier known as *EnsMart*[52], provides a web and API interface to download data such as GO terms, genes, transcripts and expression arrays from different databases using filters. *BridgeDb*[53] provides an interface to connect bioinformatics tools such as Cytoscape, PathVisio, or WikiPathways with other mapping services such as Ensembl, PICR (Protein Identifier Cross-Reference services) [54], and any local database or text files. It is intended to be used by bioinformatics developers and works on the novel idea of mapping custom identifiers to established identifiers such as Ensembl ID and then relies on Ensembl to provide the rest of the conversion. Side by side feature comparisons of these tools are provided in Table 1. Data sources for select tools are listed in Table 2.

**Table 1:** Feature comparison of different conversion tools (As of April 2012)

**Name**	**Caters**	**Intervals**	**Seqs**	**ID**	**Annot.**	**Linkout**	**Query**	**Input**	**Output**	**Annot.**	**Basis of**	**Output**	**Organisms**	**Availability**	**Last**		
	**to**	**to IDs**	**to IDs**	**lookup**	**View**		**mode**				**conversion**	**format**			**update**		
**DAVID**	probes, genes,		*✓*	*✓*	*✓*	*✓*	batch	select one	select	S, F	custom generated	html, txt	NA	web,API,	Sep, 2009		
	prots.						one						*⇓*	EASE,			
**Babelomics**	probes, genes,				*✓*	*✓*	batch	select one	select	S, F	custom generated	html, txt	11 org.	web	Sep, 2009		
	prots.						multiple										
**g:Convert**	genes , prots. and						batch	select one	select	S, F	Ensembl	html, txt, xls	H, M, R, O	web	Jun, 2011		
	probes						one										
**HMS and IC**	genes, prots. and				*✓*	*✓*	batch	select one	select	S, F	corr. files	html, txt	H, M, O	web, *⇓*	current		
	bio. molecules						one										
**Synergizer**	probes, genes and						batch	select one	select	S	Peg/custom	html, xls	H, M, R, O	web, API	May, 2011		
	Prots.						one				generated						
**Clone/Gene**	genes and					*✓*	batch	select one	select	S, F	Ensembl	html, txt, xls	H, M, R	web	Apr, 2008		
	prots.						multiple										
**ID Converter**																	
**MADGene**	probes, genes,					*✓*	batch	NA	select	S, F	MADGene link	html, xls	H, M, R, O	web, open	Aug, 2009		
	trans.						multiple						(17 org.)	source			
**GATExplorer**	Affy expression &		*✓*		*✓*	*✓*	single	probes	genes,	S	Ensembl	html	H, M, R	web, *⇓*	Mar, 2010		
	exon arrays						trans.										
**NetAffx**^**™**^	genes, prots.,				*✓*	*✓*	batch	select one	select	S, F	UniGene, LocusLink	html, txt	H, M, R, O	web	CND		
	probes, other						one										
**PLANdbAffy**	Affy expression				*✓*	*✓*	single	Affy, Hugo,Ens	Affy, Hugo,	S	RefSeq	html	H	web, *⇓*	May, 2009		
	arrays						Ens.										
**probeMatchDB**	probes, cDNA,			*✓*		*✓*	batch	select one	select	S	UniGene, Homologene	html	H, M, R	web	2006		
	EST, gene, prots.						one										
**Uniprot**	genes and				*✓*	*✓*	batch	genes or prots.	prots. or	S, F	UniProt ID	html	NA	web, API, *⇓*	Jul, 2011		
	prots.						gene										
**Onto-**	Affy, uniGene clusters,					*✓*	batch	select one	select	S, F	RefSeq, Entrez	html, email	H, M, R, O (58 org.)	web	May, 2009		
**Translate**	Acc num						one										
**AliasServer**	Affy, genes,					*✓*	batch	select one	select multiple	S	custom generated	html, txt	Not Available	Not Available	CND		
	prots.																
**MatchMiner**	Affy,			*✓*			batch	select one	choose from	S	custom generated	Email (txt,xls)	H, M	web	Sep. 2006		
	genes																
**GeneMerge**	genes and						batch	select one	NA	S, F	corr. files	html	5 org.	web	Apr, 2007		
	prots.																
**BioMart**	genes, prots.,				*✓*	*✓*	NA	select one	select multiple	S, F	NA	html, txt, xls	H, M, R, O	web, API, *⇓*	depends on DB		
	probes, other																
**BridgeDb**	probes, genes,			NA	NA	*✓*	NA	NA	NA	S, F	Ensembl, other	NA	36 org.	open source	May, 2011		
	prots., metabolites																
**AbsIDconvert**	genes, trans.,	*✓*	*✓*	*✓*	*✓*	*✓*	batch	select one	select multiple	S	Genomic Sequence	html, txt	H, M, R, O	web, *⇓*VM	Dec, 2011		
	seqs., probes											(53 org.)					

^a^Abbreviations: Annot. View: Custom Annotation view, Annot.: Annotation (S: Structural annotation, F: Functional annotation), org.: Organisms (H: Human, M: Mouse, R: Rat, O: others), prots: proteins, Affy: Affymetrix^®;^, trans: transcripts, seqs: sequences, Ens.: Ensembl, corr: correspondence, acc: accession, bio: biological, NA: Not Applicable, CND: Could not determine, *⇓*:download Knowledgebase, VM: Virtual Machine.

**Table 2:** ID converter tools, data sources and availability

**Name**	**Data Sources**	**Webpage**
DAVID	GenBank, RefSeq, KEGG, OMIM, UniGene	http://david.abcc.ncifcrf.gov/
Babelomics	Go, KEGG, Ensembl and others	http://babelomics.bioinfo.cipf.es/
g:Convert	GO, KEGG, Ensembl, TRANSFAC, Reactome	http://biit.cs.ut.ee/gprofiler/
HMS and IC	Ensembl, GO, KEGG and others	http://biodb.jp/
Synergizer	Ensembl, NCBI, RGD, SGD, KEGG, WormBase and EcoCyc	http://llama.mshri.on.ca/synergizer/translate/
Clone/Gene ID Converter	Ensembl, NCBI, Pubmed, UCSC, KEGG, Reactome	http://idconverter.bioinfo.cnio.es/
MADGene	GEO, UniGene, Entrez and others	http://www.madtools.org/
GATExplorer	Ensembl, Affymetrix^®;^	http://bioinfow.dep.usal.es/xgate/principal.php
NetAffx^™^	NCBI, GO, KEGG and others	http://www.affymetrix.com/analysis/netaffx/
PLANdbAffy	Affymetrix^®;^, UCSC, NCBI	http://affymetrix2.bioinf.fbb.msu.ru/
probeMatchDB	UniGene, HomoloGene	http://brainarray.mbni.med.umich.edu/Brainarray/
Uniprot	GenBank, RefSeq, GO and others	http://www.uniprot.org/
Onto-Translate	Ensembl, GO, KEGG and others	http://vortex.cs.wayne.edu/
AliasServer	Ensembl, EMBL, NCBI, SGD and others	http://cbi.labri.fr/outils/alias/
MatchMiner	Affymetrix^®;^, UCSC, UniGene, Entrez, OMIM	http://discover.nci.nih.gov/matchminer/index.jsp
GeneMerge	GO, KEGG	http://genemerge.cbcb.umd.edu/
BioMart	NCBI, GO, KEGG and others	http://www.biomart.org/
BridgeDb	Ensembl and others	http://www.bridgedb.org/
AbsIDconvert	UCSC, NCBI, Ensembl, Agilent, Affymetrix^®;^ and others	http://bioinformatics.louisville.edu/abid/

## Drawbacks associated with existing approaches

**Figure 2:**
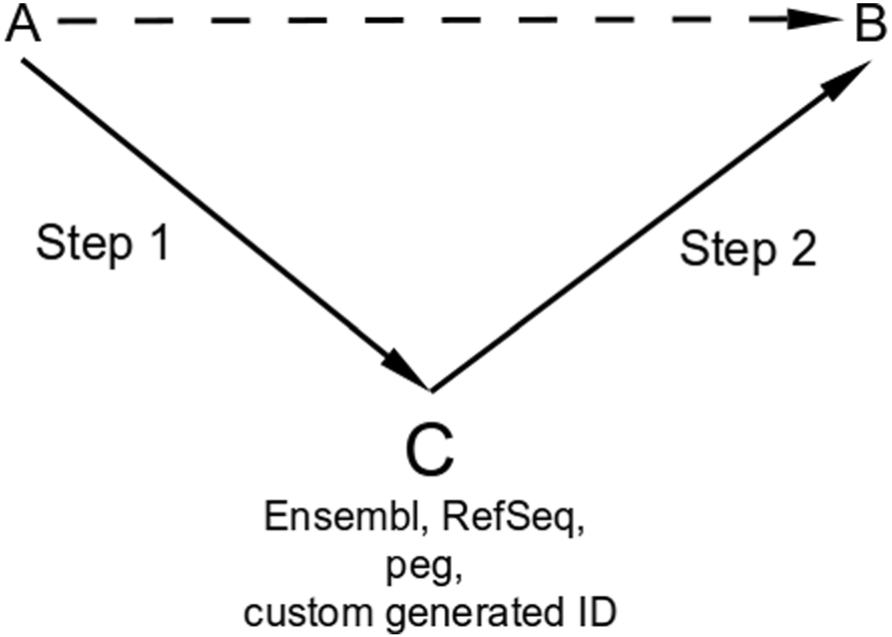
**ID conversion: A two step process Step 1: ID A is converted into ID C.** Step 2: ID C is converted into ID B

Most of the ID conversion tools mentioned above use a two step conversion method. To convert an ID “A” to ID “B”, the first step is to use a correspondence annotation relation or table to find a common intermediary ID “C” (Figure 2). This common ID “C” is then converted into target ID “B” using another correspondence table. Some tools use Ensembl or RefSeq as an intermediary while others generate unique custom identifiers. For example, the Clone/Gene ID Converter and GATExplorer use Ensembl ID, PLANdbAffy uses RefSeq whereas DAVID and Synergizer use a custom generated DAVID ID and peg respectively. These tools convert smaller fragments (probes, sequences, reads) into coarser genetic entities (Ensembl, RefSeq, EntrezID) using inferred annotation level information irrespective of the fact that these small fragments may not be representative of the annotation as a whole. These methodologies also tend to lose structural and other information available at the probe or sequence level.

As stated previously, annotations are dynamic and databases such as Ensembl and RefSeq are updated daily making it difficult to keep the databases of ID conversion tools current. This is more problematic when the intermediate IDs are custom generated as these require more effort to update. Most of the tools are based on a relational database and the dynamic nature of annotations may introduce database anomalies because of the frequent insertion, deletion and updating of the annotations. If a gene is discovered, deleted or updated in any of these databases, or the annotations corresponding to an entity are added, deleted or updated, then all the databases or correspondence tables also need to be updated. In the case of microarray experiments, if a probe corresponds to a recently deleted entity then that probe annotation needs to be edited as well. Updating any of these authoritative databases may induce a chain-reaction for any other systems using that information and any experimental result deduced from the updated probe may become invalid. Those tools that generate their own unique identifier such as DAVID, Synergizer or Babelomics, although efficient, face a similar situation and need to be updated frequently. As updating an annotation database is labor and resource intensive, some of the tools cannot afford to update their knowledgebase regularly.

## Absolute (sequence based) method for ID conversion

A feature of biological entities that is currently ignored in ID conversion is the sequence mapping information. For species where a reference genome is available, all nucleic acid and protein-based annotations, no matter the granularity, can be aligned to that reference genome sequence and therefore annotated by genomic intervals. Once the absolute genomic coordinates on a reference genome for all entities have been determined, these can be queried to find all overlapping entities, thus performing ID conversion. This conversion uses the same two step method as adopted by most of the ID conversion tools, considering the genomic coordinates as the basis of conversion, rather than the annotation level information used by other tools. Compared to other types of intermediate IDs, the intervals on a reference genome sequence are relatively static, and remapping of entities to modified genomic sequences is relatively trivial, making it possible to easily update the system. Using interval trees, conversion by finding overlapping intervals is fast and efficient [55].

**Figure 3:**
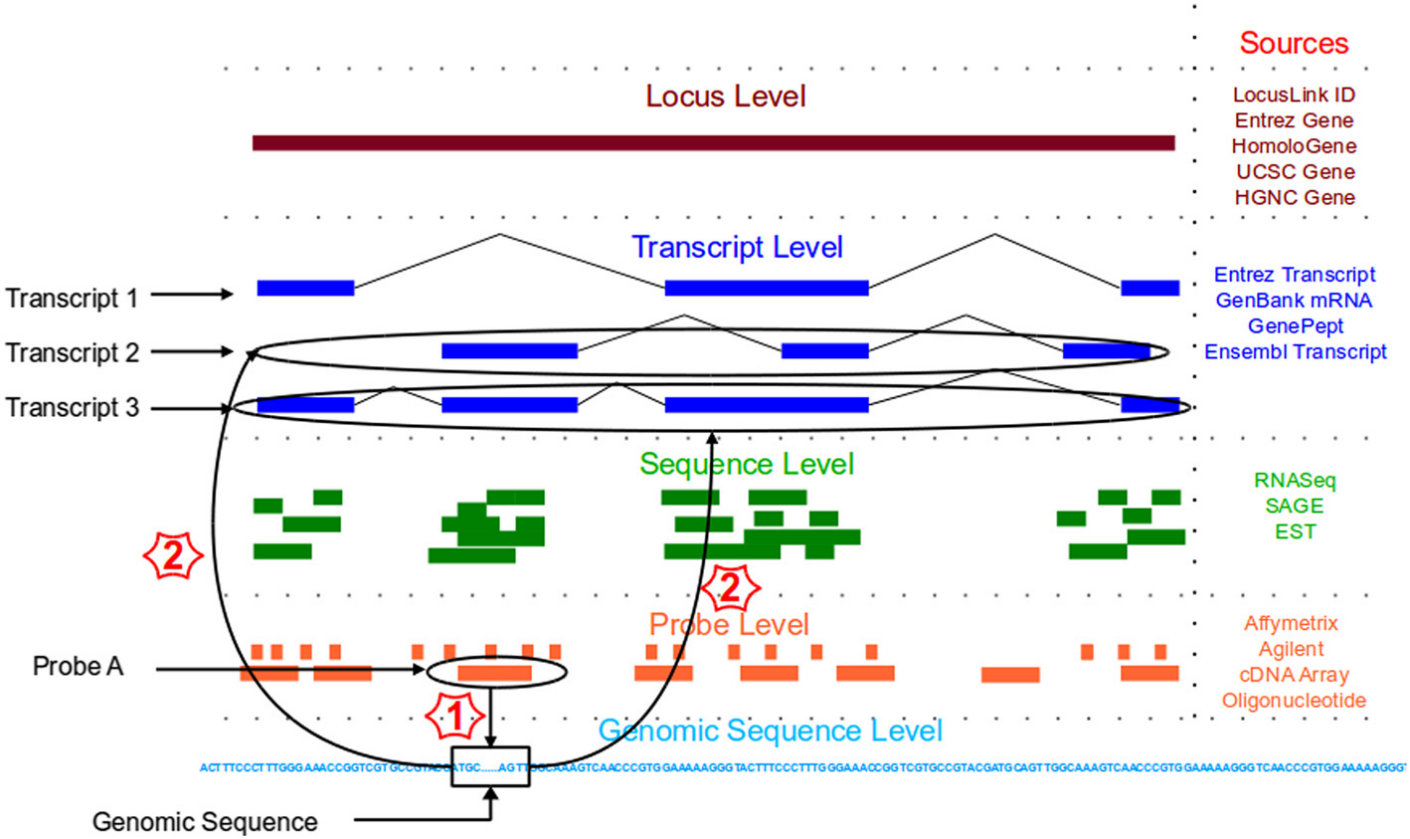
**AbsIDconvert technique.** Absolute ID conversion is a two step process whereby probe A can be converted to identifiers at the transcript level by first converting the probe to its genomic coordinates (step 1) and then determining transcripts that overlap the coordinate positions (step 2).

Figure 3 shows the steps to perform sequence-based or absolute ID conversion. In the figure, all transcripts corresponding to probe A are being found. The first step in this conversion is to find the genomic coordinates corresponding to probe A and the second step is to find all transcripts that span those coordinates. In this example transcript 2 and transcript 3 are the converted IDs corresponding to the probe A. Transcript 1 is not represented by probe A as the underlying genomic sequence is not part of transcript 1. Subsequent sections describe the design and implementation of a genomic interval based ID conversion tool, AbsIDconvert.

## Methods

**Figure 4:**
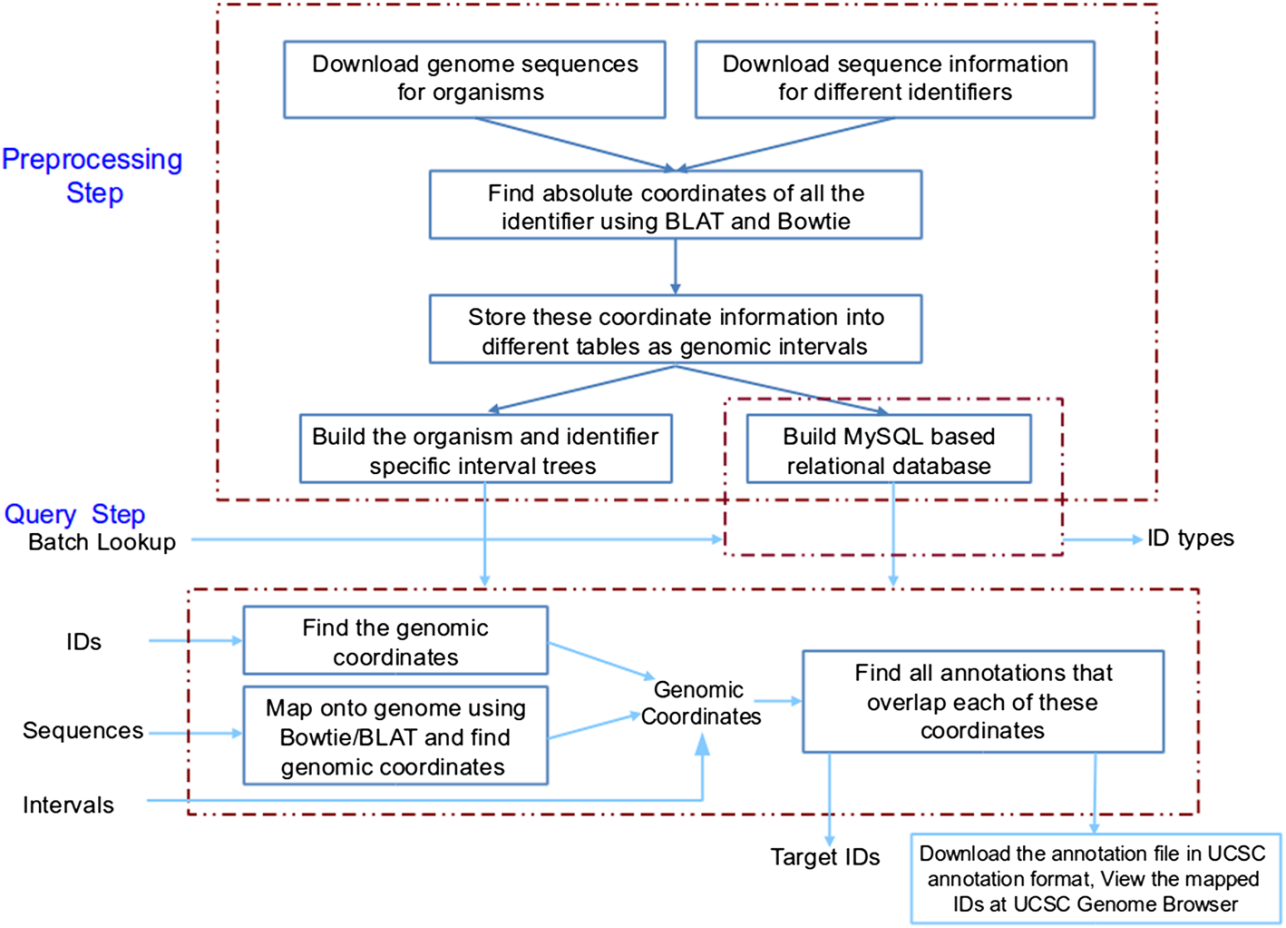
Steps involved in the construction of AbsIDconvert.

The design of AbsIDconvert was accomplished using a preprocessing and a query step. In the preprocessing step, reference genomes were downloaded from the UCSC Genome Browser (http://hgdownload.cse.ucsc.edu/downloads.html) and the NCBI website. The sequence information for a variety of identifiers at different granularities such as probes, sequences (ESTs), transcripts and genes were downloaded from their respective authoritative websites or UCSC. The identifier types include Affymetrix^®;^ probes, Agilent probes, EST sequences, Ensembl transcripts and Entrez genes. Each identifier sequence was mapped to the respective genome using either BLAT [43] or Bowtie [56]. BLAT was used to map longer (>100 BP) sequences, while Bowtie was used for relatively short (≤ 100 BP) sequences such as Affymetrix^®;^ and Agilent probes. Each identifier was then annotated with structural information such as *start* (identifier’s start coordinate on genome), *end* (the end coordinate on the genome), *size* (sequence size) and *chrom* (corresponding chromosome). This information was collected for each identifier as a genomic interval. Genetic entities with multiple exons such as transcripts were treated differently as there are two ways in which these can be structurally annotated. One method is to use the extreme ends (i.e. start and end codons of the transcript) as their intervals including both the exons as well as intronic regions, or alternatively exclude the intronic regions and assume the transcript’s genomic intervals are an assembly of genomic intervals of the participating exons (AbsIDconvert incorporates both). Finally organism and identifier type specific interval trees were constructed and stored. A list of all identifiers and their type was also stored in a relational database to facilitate batch look-up for the types of identifiers. Figure 4 shows the design steps of AbsIDconvert.

Once structural annotation for each of the identifiers is available, AbsIDconvert can query this information. This query step uses the structural annotation information of each identifier and the organism specific database generated from the previous step. AbsIDconvert assumes two biological entities (nucleic acid, protein entity) are the same if their genomic sequences are also the same, overlap or one is contained within the other. As the number of annotations are large and frequent insertions and deletions are routine, an efficient data structure for storage and computational operations is needed. Considering that the structural annotation is in the form of genomic intervals, a modified Red-Black tree, known as an interval tree, is used to store the information for all IDs. An interval tree maintains a dynamic set of elements, with each element *x* containing an interval *int[x]*. This *int[x]* stores the start and end of the interval apart from other auxiliary information. This data structure is dynamic in nature and can perform insertions and deletions efficiently in time *O*(*log*_2_*n*), where *n* is the number of elements. Interval trees have been shown to be efficient for working with a large number of genomic intervals [55].

**Figure 5:**
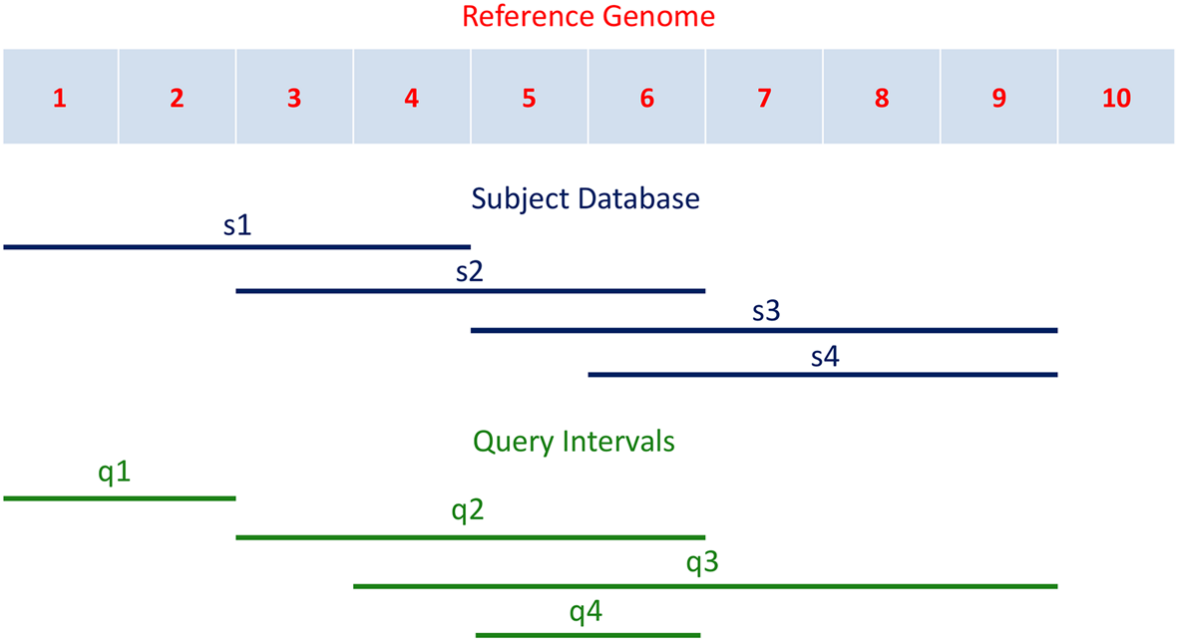
**Example of interval overlaps.** The reference region is 10 bases in length, with database annotations s1–s4. Queries q1–q4 are used to obtain the corresponding annotations.

There are four possible ways in which AbsIDconvert may be queried: 

• Lookup identifiers: Given a mixed list of identifiers, AbsIDconvert can determine the types of identifiers in the list. This step uses the relational database created in the preprocessing step and can efficiently categorize the IDs in the list.

• Batch conversion of IDs: Given a list of identifiers, AbsIDconvert uses the interval tree to find their genomic coordinates. Once the coordinate information is available, all overlapping identifiers can be found by querying the interval tree. This uses the IRanges [57] and GenomicRanges [58] packages internally to maintain the genomic intervals which are based on Allen’s Interval Algebra [59]. Users can specify various range parameters using the interface. The overlap type (‘type’) parameter may take any one of ‘any’, ‘start’, ‘end’, ‘equal’ or ‘within’ as its value. By default ‘any’ overlap is accepted. If ‘type’ value is ‘start’ or ‘end’ then the query intervals are required to have matching ‘start’ and ‘end’ respectively with subject intervals in the database. If ‘type’ is ‘equal’ then only those subjects are retrieved which have the exact same coordinates. For ‘within’, the query must be contained wholly within the subject intervals. Another parameter is for specifying the maximum gap (‘maxgap’) between subject and query intervals to consider them as overlapping. The default value is zero which assumes there should not be any gap between the subject and query intervals. This parameter is useful for finding genes in the flanking regions of the specified intervals. The third parameter is the minimum overlap (‘minoverlap’) size that specifies the minimum number of overlapping base pairs needed to consider the query and subject an overlap. The default overlap value is one. The last parameter is the ‘select’ parameter that specifies which type of overlaps will be reported. By default, all overlapping intervals will be reported. Selecting ‘first’, ‘last’ and ‘arbitrary’ will report first, last and arbitrary overlapping intervals from the result. A simple example using intervals is shown in Figure 5. In this case, the reference genome is 10 BP long. The subject database contain four intervals s1, s2, s3 and s4 that represent the interval database. Query intervals also consist of four intervals q1, q2, q3 qnd q4. Considering default values for range parameters, q1 overlaps with s1, q2 and q3 overlap with all the intervals in the subject, whereas q4 overlaps with s2, s3 and s4. If the values of the parameters are type=‘within’, maxgap = 0, minoverlap=1, select= ‘all’ then q1 overlaps with s1, q2 with s2 and q4 with s2 and s3. If the values of the parameters are type=‘end’, maxgap = 1, minoverlap = 1, select= ‘all’ then q2 overlaps with s2, q3 with s3 and q4, and q4 with s2.

• Intervals as input to AbsIDconvert: A unique feature of the ID conversion is to find target identifiers corresponding to a given interval. For example, next-generation sequencers generally map the DNA sequences or reads to a reference genome and output the intervals for each aligned reads. Finding desired target identifiers corresponding to these intervals is routinely required. AbsIDconvert efficiently converts these coordinates into target identifiers in a high throughput manner. For instance, a user of AbsIDconvert is able to take a set of intervals upstream of a set of transcription start sites to determine if any features are annotated proximal to the regions of interest.

• Sequences as input to AbsIDconvert: Sometimes a user may be interested in finding all identifiers that correspond to a particular sequence or a list of sequences. For instance, a user may be interested in finding all gene names and Entrez IDs corresponding to a set of sequences. In this case, AbsIDconvert maps these sequences to the corresponding genome (or any other genome for cross–species comparisons) and determines the genomic intervals they belong to and then retrieves all the desired target identifiers that overlap these intervals. Due to the computational complexity involved in mapping long sequences using a generic mapping algorithm such as BLAT or BLAST, the web version of AbsIDconvert supports only short sequence mapping using Bowtie. Longer sequences can be mapped using BLAT in the virtual machine version of AbsIDconvert. Sequence output from next-generation sequencing technologies can be catered efficiently using AbsIDconvert. Alternatively, the coordinate information may be obtained by submitting the sequences to Galaxy [60-62] or the UCSC genome browser and subsequently inputting the intervals using AbsIDconvert. Mapping parameters can be specified by the user through the interface. Parameters include the maximum number of mismatches which can range from zero (default) to three. The second mapping parameter specifies which type of alignments are to be reported. The default value is ‘all Best’ in which all best alignments will be reported by Bowtie. However, ‘all’, ‘k’ or ‘k Best’ can be selected for Bowtie output. AbsIDconvert also has another parameter ‘Do not report (.more)’ that takes a positive integer value which specifies that Bowtie will suppress all alignments for a particular read if the total number of reportable alignments for that read is more than the specified value. The default value of -1 specifies that all alignments will be accepted. For instance, if this value is set to 100, then Bowtie will suppress all those alignments for reads that map to 100 or more locations on the genome. This is an effective option to mask repeat sequences or small sequences from appearing into the output because their probability to map at multiple locations on the genome is higher.

AbsIDconvert supports 53 major species for performing ID conversion on a list of identifiers and a list of intervals. It also has sequence level mapping support for 12 major species including *Homo sapiens, Mus musculus, Rattus norvegicus, Bos taurus, Gallus gallus, Sus scrofa, Xenopus tropicalis, Anopheles gambiae, Drosophila melanogaster, Caenorhabditis elegans, Saccharomyces cerevisiae*, and *Danio rerio*. AbsIDconvert converts the input (intervals, IDs and sequences) into target identifiers with links to authoritative databases. All intermediate interval files are available to download for later use. It also generates custom annotation files that can be used to view the IDs simultaneously (chromosome–wise) as a custom track in the UCSC Genome Browser. The performance and potential uses for AbsIDconvert are discussed in the following sections.

## Results and discussion

### Intervals vs. relational database

The genomic coordinate information for different identifier types mapped to 53 species were stored as intervals. An interval tree method was implemented and used to store and query the corresponding interval information for each identifier type. For comparison with relational databases, an equivalent MySQL database was implemented to perform ID conversion based on coordinate information, and the run time for both of these methods were compared.

**Figure 6:**
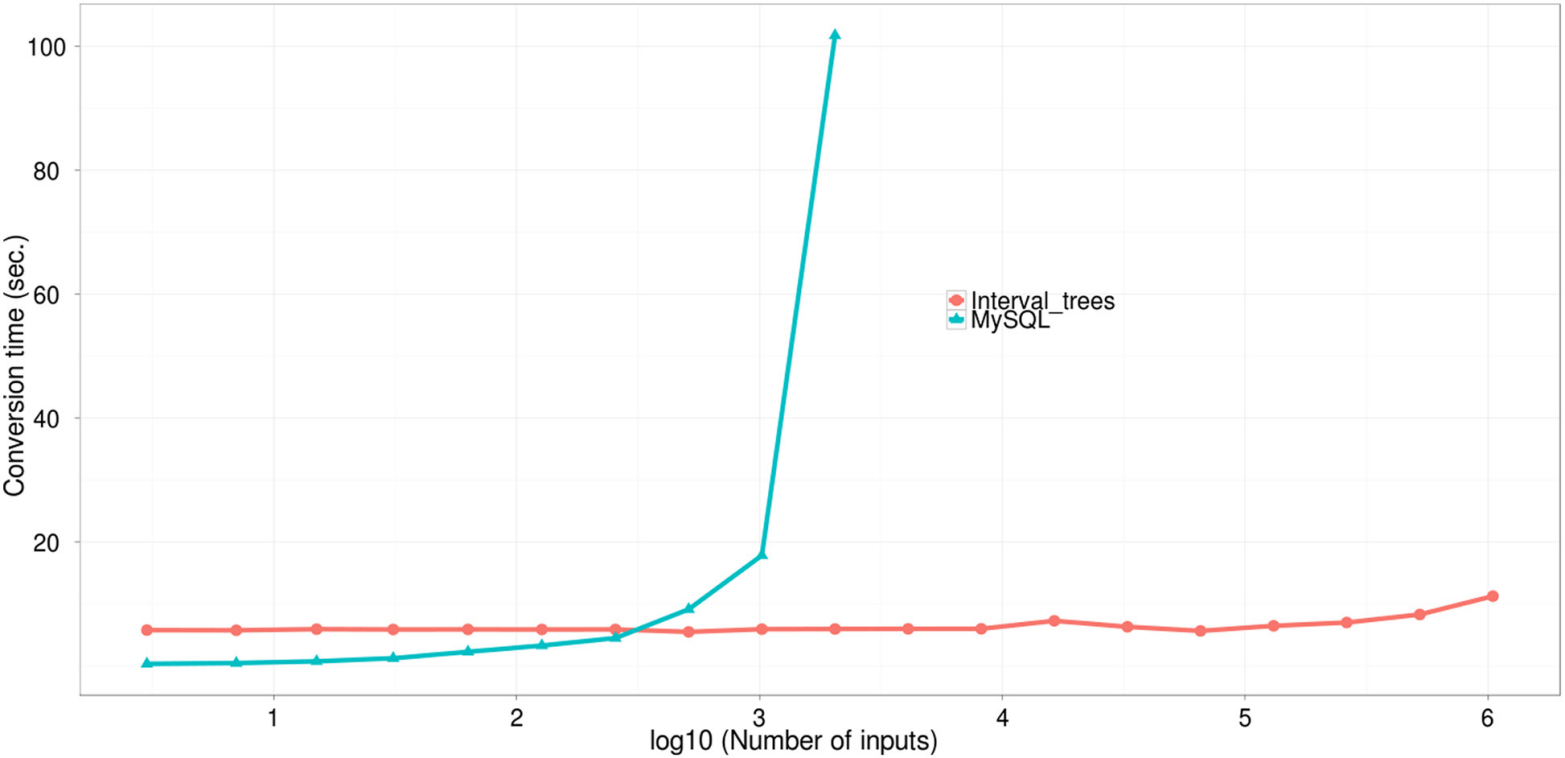
Runtime comparison between MySQL and interval-tree approach while converting EST IDs into Entrez Gene IDs.

Run–time comparisons of the interval tree and MySQL implementations were performed using randomly sampled rat EST IDs which were subsequently converted to Entrez gene IDs. To test the actual runtime, the number of EST IDs was increased exponentially for each test point and the corresponding execution time (in seconds) was measured. The run time complexity of the interval tree maintained a constant rate while the relational methodology grows in linear fashion, allowing for the conversion of millions of identifiers in only a few seconds (Figure 6).

Further analysis of conversion runtime was performed using 1,000 random sampled IDs from Affymetrix^®;^ Rat230_2 microarray probes, Agilent Cgh105a microarray probes, RefSeq IDs, Ensembl transcripts, Entrez genes, HUGO gene symbols and EST IDs which were converted into one another using the web version of AbsIDconvert (Table 3). The extreme left column represents the source identifiers which are converted to target identifiers shown in first row. The numbers in small parentheses in the first row show the total number of genomic coordinates for individual ID types (for instance, Affymetrix^®;^ Rat230_2.0 probes have altogether 231,971 intervals stored). Since AbsIDconvert supports conversion to multiple target types, the last column represents the time elapsed when an input type is converted into all other ID types.

**Table 3:** Run time (sec.) to convert 1,000 IDs from one type to another using web–based AbsIDconvert

	**Rat230_2**	**Cgh105a**	**RefSeq**	**EnsTrans**	**Entrez gene**	**GeneSymbol**	**EST seq**	**All**
	**(231,971)**	**(97,973)**	**(160,644)**	**(349,445)**	**(30,972)**	**(30,972)**	**(3,918,403)**	
Affymetrix Rat230_2	5.6	3.2	4.1	7.6	3.2	3.3	33	47.6
Agilent Cgh105a	5.1	3.9	2.5	2.7	2.92	3.05	31.3	55.6
RefSeq	4.5	3.1	3.6	3.6	2.3	2.2	31.9	34.5
Ensembl transcript	2.9	3.8	3.1	4	2.47	3.02	34.6	47.1
Entrez gene	2.7	2.9	2.8	3	7.5	7.1	18.4	35.3
Gene symbol	2.9	2.8	2.7	2.9	8.5	7.5	16.6	38.2
EST sequences	18.6	17.6	31	30.3	28.3	29.3	64.1	73.7

### Run–time comparison

**Figure 7:**
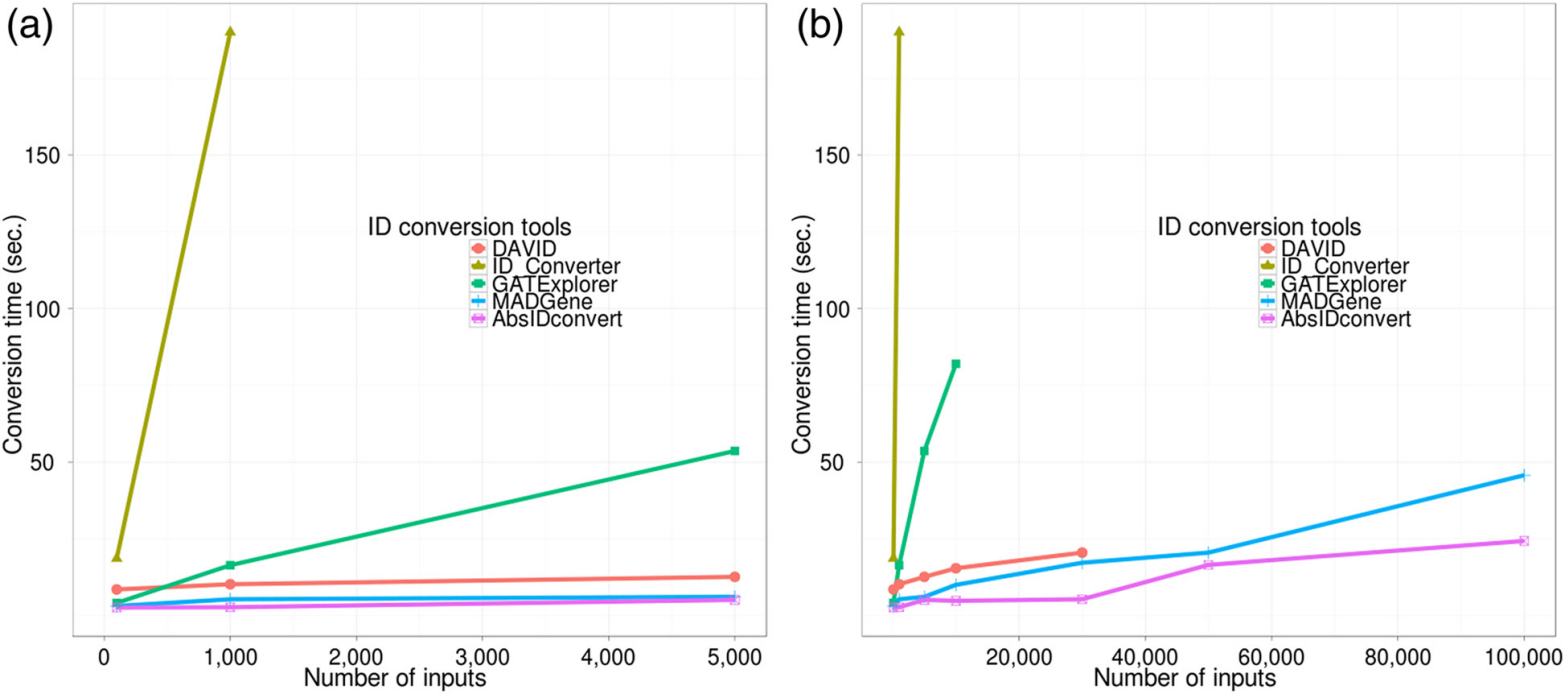
**Runtime comparison of AbsIDconvert with other conversion tools.** (**a**) Tools give comparative run times with a small input (i.e. ≤5,000). (**b**) The number of inputs was gradually increased to 100,000 and run times for each tool were determined. Most of the tools were not able to produce the result for large inputs. Only MADGene and AbsIDconvert could produce results. Note that DAVID limits user input to 30,000 identifiers.

Direct comparison to other ID conversion approaches is not straightforward due to the differences in annotation information (based on the last available update), supported ID types, and development/deployment platforms. In order to test the runtime of comparable solutions (DAVID, Clone/Gene ID Converter, GATExplorer, MADGene, and AbsIDconvert), a varying number (100 to 30,000) of Affymetrix^®;^ Rat230_2.0 microarray probesets were converted to Entrez IDs (Figure 7). When the number of probe sets converted was small (100), the conversion time for all tools was nominal. For a moderate number of probe sets (5,000) MADGene, DAVID and AbsIDconvert performed similarly (12.6, 6.1 and 5.1 sec. respectively), while GATExplorer took around a minute and Clone/Gene ID Converter took 15 minutes (Figure 7(a)). As the number of probe sets further increased, all of the tools, with the exception of MADGene and AbsIDconvert, were incapable of tractably handling such a large number of inputs. Since the Affymetrix^®;^ Rat230_2.0 has roughly 31,000 unique probe sets and over 300,000 individual perfect match probes, a run time comparison for a large number of inputs (>30,000) was performed by converting randomly sampled human transcripts into Entrez IDs (direct conversion of individual probes is not possible within all of the tools; therefore the closest comparison is made to the same number of human transcripts). For 100,000 inputs, only MADGene and AbsIDconvert completed successfully, taking 45 sec and 24 sec, respectively (Figure 7(b)). The run–time complexity for AbsIDconvert compares favorably to other similar tools, demonstrating its applicability in the analysis of high throughput data.

### Output accuracy

The accuracy of conversions performed using AbsIDconvert was assessed based on the overlap of the successfully converted IDs with those found using other tools for three types of conversions. In the first conversion, 1,000 unique Entrez IDs were randomly sampled from the “org.Hs.eg.db” Bioconductor annotation package and converted to their corresponding official gene symbols. Ten ID conversion tools, from a total of 19 tools listed in Table 1, can perform this conversion. Considering NCBI as the authority for Entrez IDs, the accuracy of different conversion tools were evaluated using the following assumptions: 

1. NCBI contains the most up to date information and its annotations are correct.

2. An Entrez ID may be annotated by more than one gene symbol.

3. Given an Entrez ID *x*, if a tool converts *x* to a set of gene symbols, *Y* (*x *→* Y*), and NCBI annotates *x* to another set of gene symbols, *Z* (*x*→*Z*), then accuracy terms can be defined as:

• **True positives (TP)** are those conversions in which the converted gene symbol set contains all the gene symbol(s) annotated by NCBI (i.e. *Z *⊆* Y*).

• **False positives (FP)** are unexpected results. This includes incorrect conversions (*Z *⊈* Y*), as well as those conversions in which NCBI does not annotate an Entrez ID with any gene symbol, but a tool finds some gene symbol corresponding to that Entrez ID (*Z *=* ϕ *and *Y *≠* ϕ*).

• **False negatives (FN)** are missing conversions in which a tool could not find corresponding gene symbol(s) (*Z *≠* ϕ *and *Y *=* ϕ*).

• **True negatives (TN)** are the correct absence of conversion in which NCBI as well as a particular tool does not convert an Entrez to any gene symbol (*Z *≠ *ϕ *and *Y *≠* ϕ*).

4. Accuracy is defined as

(1) $$\%\text{Accuracy}(\text{ACC})=\frac{\text{TP}+\text{TN}}{\text{TP}+\text{TN}+\text{FP}+\text{FN}}\times 100$$

**Figure 8:**
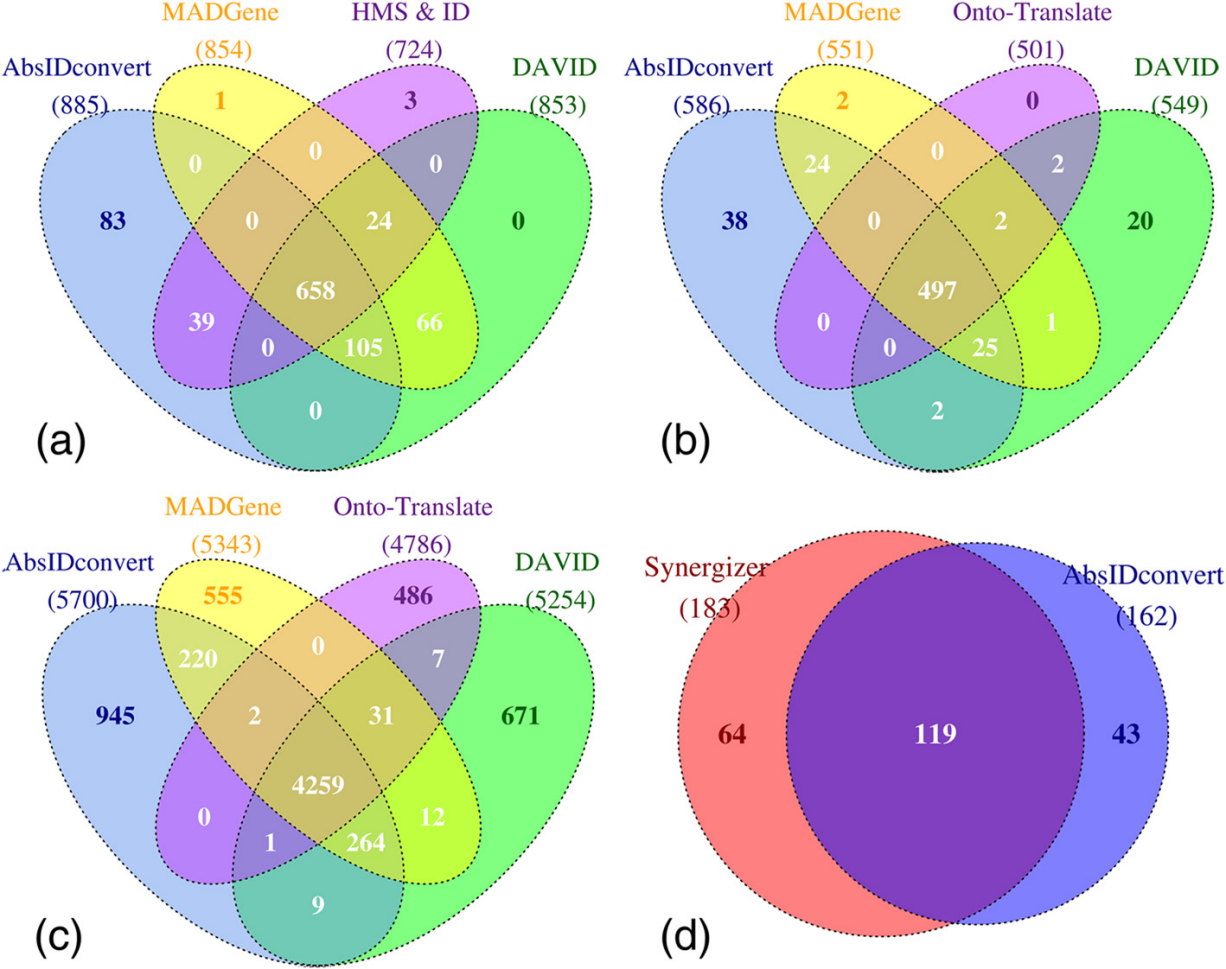
**Venn diagram showing conversion results for the top performing conversion tools.** (**a**) Entrez IDs converted to official gene symbols. (**b**) Entrez IDs converted to RefSeq IDs. (**c**) Entrez IDs converted to RefSeq IDs using cumulative bootstrap. (**d**) Affymetrix^®;^ HG_U133Plus2.0 probesets converted to Agilent Cgh44b probes.

Table 4 shows the contingency table and associated statistics for the conversion of 1,000 Entrez IDs to gene symbols. AbsIDconvert converted a total of 885 Entrez IDs with an accuracy of 87.2% followed by DAVID (853, 79.1%), MADGene (854, 73.1%) and HMS & IC (724, 72.9%). Although Onto-Translate converted a total of 823 Entrez IDs, it has more FP conversions than HMS & IC and therefore a lower accuracy. We further investigated the conversions from the top four tools on the basis of their accuracy and summarized the results in a Venn diagram (Figure 8(a)). AbsIDconvert converted a total of 83 Entrez IDs which are missed by the other tools. NCBI places all these Entrez IDs onto the reference genome and annotates them with gene symbols that are in agreement with AbsIDconvert (Additional file 1). Of these 83 Entrez IDs, 48 are categorized as “pseudo”, 27 as “miscRNA”, four as “protein-coding”, three as “unknown” and one as “other”. AbsIDconvert was unable to convert a total of 115 Entrez IDs, out of which 21 IDs were not converted by any of the tools examined.

**Table 4:** Entrez ID to gene symbol conversion accuracy

**Tool**	**totalMapped**	**TP**	**FP**	**FN**	**TN**	**TPR**	**FPR**	**ACC**	**FDR**	**F1_score**
AbsIDconvert	885	866	19	109	6	88.82	76.00	**87.20**	2.15	93.12
DAVID	853	790	63	146	1	84.40	98.44	**79.10**	7.39	88.32
MADGene	854	730	124	145	1	83.43	99.20	**73.10**	14.52	84.44
HMS & IC	724	723	1	270	6	72.81	14.29	**72.90**	0.14	84.22
Onto-Translate	823	722	101	176	1	80.40	99.02	**72.30**	12.27	83.90
MatchMiner	539	457	82	458	3	49.95	96.47	**46.00**	15.21	62.86
Clone/Gene ID converter	537	441	96	457	6	49.11	94.12	**44.70**	17.88	61.46
g:Convert	445	433	12	549	6	44.09	66.67	**43.90**	2.70	60.69
Synergizer	445	433	12	549	6	44.09	66.67	**43.90**	2.70	60.69
Babelomics	486	421	65	508	6	45.32	91.55	**42.70**	13.37	59.51

Of the 94 Entrez IDs that AbsIDconvert was not able to convert but other tools were (Additional file 2), most were either “not on current assembly”, meaning that the reference sequence for that Entrez ID could not be mapped to the current genome (28 IDs), but could be mapped to previous genome assemblies; or “not annotated on reference assembly”, indicating that the sequence cannot be found on the reference assembly at all (61 IDs). Five conversions were found where the Entrez IDs reported had since been deleted and replaced (DAVID and MADGene both converted these IDs).

In a second conversion test, 1,000 randomly sampled Entrez IDs were converted to RefSeq IDs using ten of the 19 tools listed in Table 1 (the others are not able to perform this type of conversion and were not evaluated). There are many different classes of RefSeq IDs, including mRNA (ID starts with NM_ ), RNA (NR_ ), protein (NP_ ), as well as predicted versions of each one (XM_ , XR_ and XP_ respectively). How RefSeq IDs are segregated for conversion differs among the tools tested. For example, a number of tools combine all the different types of RefSeq IDs into one converted ID type while others treat each one separately. Other tools ignore the predicted RefSeq IDs and only consider mRNA and RNA. For example, AbsIDconvert’s RefSeq database combines both mRNA and RNA, whereas MADGene includes predicted products (XM). DAVID and Synergizer have separate options for RNA and mRNA RefSeq. Therefore, to enable comparison between all the tools, only those conversions that result in mRNA or RNA RefSeq IDs are considered, and for those tools that report them separately, the results from both conversions were combined. In addition, any predicted RefSeq IDs (i.e. those that begin with *X*) were removed.

Using the same assumptions as reported for the Entrez to Symbol conversion, the accuracy of conversion for each tool was calculated (Table 5). Of the 1,000 Entrez IDs used, NCBI annotates only 599 with one or more RefSeq. In this case, the accuracy for the various tools ranged from a high of 75.6% (AbsIDconvert) to a low of 38.9% (HMS & ID).

**Table 5:** Entrez ID to RefSeq ID conversion accuracy

**Tool**	**Total Mapped**	**TP**	**FP**	**FN**	**TN**	**TPR**	**FPR**	**ACC**	**FDR**	**F1_score**
AbsIDconvert	586	362	224	20	394	94.76	36.25	**75.60**	38.23	74.79
MADGene	551	335	216	49	400	87.24	35.06	**73.50**	39.20	71.66
Onto-Translate	501	291	210	99	400	74.62	34.43	**69.10**	41.92	65.32
DAVID	549	311	238	72	379	81.20	38.57	**69.00**	43.35	66.74
Synergizer	482	278	204	121	397	69.67	33.94	**67.50**	42.32	63.11
g:Convert	482	278	204	121	397	69.67	33.94	**67.50**	42.32	63.11
MatchMiner	474	268	206	126	400	68.02	33.99	**66.80**	43.46	61.75
Babelomics	501	267	234	128	371	67.59	38.68	**63.80**	46.71	59.60
Clone/Gene ID converter	421	219	202	195	384	52.90	34.47	**60.30**	47.98	52.46
HMS & ID	461	227	430	181	162	55.64	72.64	**38.90**	65.45	42.63

The results from the four most accurate tools were investigated further. 497 Entrez IDs were converted commonly by all tools (Figure 8(b)). AbsIDconvert converted 586, followed by MADGene (551), DAVID (549) and Onto-Translate (501). Five conversions specific to MADGene were not found by AbsIDconvert (Additional file 3). In this case, AbsIDconvert correctly mapped the Entrez IDs to the genome (Additional file 4); however, the corresponding RefSeq IDs were not in the data obtained from UCSC. Other conversions that AbsIDconvert did not report were found to be false positives reported by other tools. For example, DAVID and Onto-Translate both reported converting “4586” to “NM_017511” and “441956” to “NM_001013729”; however, the genomic intervals for those IDs do not overlap, and both RefSeq IDs are shown in NCBI as “permanently suppressed”. For the twenty conversions specific to DAVID, the reported RefSeq IDs were found to be associated with different Entrez IDs in NCBI (Additional file 5).

The thirty-eight Entrez IDs converted only by AbsIDconvert were investigated further to verify whether they were “correct”. Thirty-three are in agreement with the NCBI data (Additional file 6). For the other five, we examined the genomic intervals of both the Entrez IDs and reported RefSeq IDs to verify that they do indeed overlap (intervals are reported in Additional file 7). In all cases the converted IDs do have overlapping intervals with two of the Entrez IDs discontinued and replaced since the initial construction of the AbsIDconvert database, “100505905” (to “23189” on March 2, 2012) and “100652874” (to “100505641” on Feb 3, 2012).

To better assess the accuracy of AbsIDconvert compared to other tools, the Entrez to RefSeq ID conversion was repeated ten times, randomly choosing 1,000 Entrez IDs each time. Out of the 10,000 randomly selected Entrez IDs, 8,974 were unique. AbsIDconvert converted 5,700 (63%), followed by MADGene (5,343, 59.5%), DAVID (5,254, 58.5%) and Onto-Translate (4,786, 53.3%) (Figure 8(c)). A total of 945 (10%) of the IDs were exclusively converted by AbsIDconvert.

In the third conversion, 1,000 randomly sampled human Affymetrix^®;^ GeneChip HG-U133 Plus 2.0 probesets were converted to Agilent Cgh44b probes (Figure 8(d)). This type of cross-platform conversion is important in meta-analysis studies where results are drawn by integrating and analyzing data from a number of independent studies/platforms. As this type of conversion is available only in Synergizer, we compared the conversion results of this tool with AbsIDconvert. Synergizer converted 183 whereas AbsIDconvert converted 162 probesets. The reason for the small number of conversions is primarily due to the design differences of the probes on these chips. Two questions required deeper investigation: 1. Why was AbsIDconvert not able to convert 64 Affymetrix^®;^ IDs that were successfully converted by Synergizer; and 2. Are the 43 conversions exclusive to AbsIDconvert valid? To answer these, we extracted the design annotation of all the Affymetrix^®;^ GeneChip HG-U133 Plus 2.0 probesets provided by Affymetrix’s NetAffx [63] along with the design annotations for the Agilent Cgh44b probes supplied by Agilent [64]. These provided the individual locations of each probe on the hg19 genome, thereby enabling investigation of the interval separation between the probesets.

In order to examine the 64 probesets converted by Synergizer but not by AbsIDconvert, the genomic location(s) of the Affymetrix^®;^ probesets were compared to the genomic locations of the Agilent probes. Fifty-six (out of 64) of the probes are separated according to their genomic locations and do not overlap at all. This separation ranges from 75 to 418,671 BP with a median separation of 4,736 bases. Further analysis determines that these all lie in the regions between the individual probes of the respective probesets and therefore have no shared sequence identity.

Most of the ID converter tools including Synergizer map the genetic entities (probes, probesets) spanning tens of bases to an intermediary such as Ensembl that is at a coarser granularity spanning a few kilobases with possible intronic regions. While performing conversions, these tools only use the probe annotation, disregarding the actual sequence information. The above false positives provided by Synergizer are likely the result of ignoring the sequence level information as the two types of probes actually span different genomic intervals.

Next we considered conversions found exclusively by AbsIDconvert. Based on the official annotation from NetAffx™, we found that intervals for all 43 Affymetrix^®;^ probesets actually contain or overlap the converted Agilent probes with a mean overlap of 56.43 bases. Considering that most of the Agilent probes are 60 bases long and an Affymetrix^®;^ probeset contains overlapping 25 bp probes, this indicates most of these Agilent probes are contained in the Affymetrix^®;^ probeset region. These probesets were checked at the probe level and it was determined that these converted Agilent probes overlap with individual Affymetrix^®;^ probes to some extent, or are completely contained with a mean overlap length of 38.70 BP. We are not sure why Synergizer was unable to convert these 43 probes; however, the official annotation confirms these annotations and bolsters our confidence in the power and accuracy of our sequence based ID conversion.

## Case studies

Three illustrative case studies were explored to demonstrate the capabilities of AbsIDconvert. The first case study considers sequence-based mapping of identifiers in a comparative genomics analysis of organisms involved in malaria; the second examines remapping of probes to annotations within and across species using a historical cDNA platform from Incyte; and the third identifies Ensembl transcripts mapped by Agilent and Affymetrix^®;^ arrays.

### Case study 1: Comparative genomics: plasmodium mapped to human and *Anopheles gambiae*

Recent studies have surveyed the role of both host and pathogen genetic variability to determine molecular signatures for host-pathogen interactions [65]. While the interactions between a pathogen and its host are often mediated by the host immune system responses to the pathogen, host-pathogen relationships theoretically have the potential to create a metagenomic environment whereby the total transcriptome is contributed by both the host and pathogen genes. In some cases, such as *Neisseria meningitidis*, a direct interaction between host and pathogen genes has been demonstrated [66]. As an illustrative example, it might be possible that shared sequence similarities between pathogen and host genes play a role in host gene regulation via pathogen genes and gene products that provide additional promoter sites, miRNA targets, and binding motifs similar to those found in the host. To test the feasibility of this possibility in the context of malaria, we used absIDConvert to identify coding sequences identical between the *Plasmodium falciparum (PF)* and *Plasmodium vivax (PV)* species and the human and anopheles genomes.

Plasmodium is a parasite responsible for causing malaria in humans primarily in tropical and sub–tropical areas. About 3.3 billion people are at risk of this disease, leading to 250 million malaria cases and one million deaths worldwide every year (http://www.who.int/features/factfiles/malaria/). Altogether four Plasmodium species are responsible which are carried by the female *Anopheles gambiae* mosquito. PF and PV are the most common, with PF being the deadliest.

**Figure 9:**
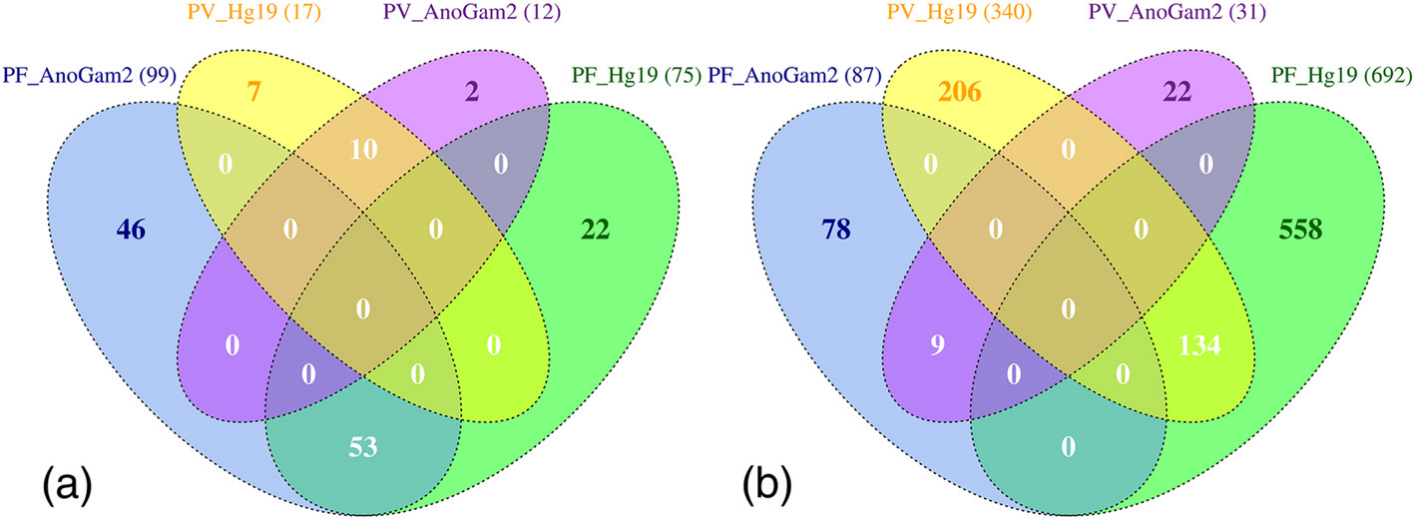
**Case study 1 - Comparative genomics study using AbsIDconvert. (a)** Venn diagram showing the number of gene fragments from *P. falciparum* (PF) and *P. vivax* (PV) which overlaps with at least one gene from *Anopheles gambiae* and *Homo sapiens*. **(b)** Corresponding genes in *Anopheles gambiae* (AnoGam2) and *Homo sapiens* (hg19) that were mapped by gene fragments from *P. falciparum* and *P. vivax*. Only those genes were considered which had the exact same sequence as the gene fragments.

Coding sequences for each gene for these two species were downloaded from the PlasmoDB website (http://plasmodb.org/) [67]. The total number of coding sequences in PF and PV were 5,524 and 5,435 respectively. Sequences for each of these genes were then fragmented into 50 base-pair (BP) long sequences with an overlap of 25 BP. The fragmented sequences were given a unique name by attaching a numerical suffix onto the gene name that denotes the order of appearance in the gene sequence. These fragmented sequences were analyzed using AbsIDconvert by selecting default parameters including no mismatch while aligning to the *Anopheles gambiae* (AnoGam2) and *Homo sapiens* (hg19) genomes (Figure 9).

A total of 75 gene fragments from PF (PF_Hg19 in Figure 9(a)) had an exact sequence match to 692 human genes (PF_Hg19 in Figure 9(b)). For PV, the aligned number of gene fragments and corresponding genes were 17 (PV_Hg19 in Figure 9(a)) and 340 (PV_Hg19 in Figure 9(b)), respectively. These numbers indicate that the gene fragments align to multiple locations on the human genome. Among genes that were mapped from PF and PV gene fragments, a total of 134 genes were common. When the same gene fragment sequences from PF and PV were aligned to the *Anopheles gambiae* genome (AnoGam2), a total of 99 (PF_AnoGam2 in Figure 9(a)) gene fragments from PF were mapped to 87 (PF_AnoGam2 in Figure 9(b)) different genes, showing that the correspondence between the gene fragments and genes is largely one–to–one. These numbers for PV were 12 (PV_AnoGam2 in Figure 9(a)) and 31 (PV_AnoGam2 in Figure 9(b)), respectively.

A more detailed analysis of the genes identified using ontological information indicates a significant enrichment in cell adhesion processes (Table 6). These are present in the GO terms ‘cell-cell adhesion’ (and others), but also implied by the large number of terms regarding neuronal axonogenesis and synapse formation, which require specific regulation of cellular adhesion. While purely speculative at this point, it is possible these plasmodium genes interact with the human host to help sequester human erythrocytes in small blood vessels which aids in the invasion plasmodium into the immune system [68]. While benchtop analysis of these genes is needed to determine if the “feasible” actually occurs, it is clear that analysis using AbsIDconvert has identified, via cross-species analysis, a limited set of genes that can be further interrogated for understanding the malaria-related pathophysiology, including the process of plasmodium incorporation into erythrocytes.

**Table 6:** **Significantly enriched (p-value < 0.001, number of genes ≥ 2) Gene Ontology biological processes for the *P. falciparum *and *P. vivax *genes**

**GO ID**	**Description**	**listMembership**	**pFal.Pvalue**	**pViv.Pvalue**
GO:0048639	positive regulation of developmental growth	pFal	0.00023	0.078421
GO:0051865	protein autoubiquitination	pFal	0.000611	0.310842
GO:0007417	central nervous system development	pFal	0.000749	0.052751
GO:0010559	regulation of glycoprotein biosynthetic process	pFal	0.000534	0.189699
GO:0043062	extracellular structure organization	pFal	0.000896	0.056366
GO:0031290	retinal ganglion cell axon guidance	pFal	0.000729	0.020543
GO:0050772	positive regulation of axonogenesis	pFal	0.000671	0.108078
GO:0007268	synaptic transmission	pFal	9.63E-005	0.004437
GO:0007156	homophilic cell adhesion	pFal	2.90E-005	0.00181
GO:0048745	smooth muscle tissue development	pFal	0.00097	0.215514
GO:0008038	neuron recognition	pFal,pViv	0.000611	2.71E-005
GO:0071702	organic substance transport	pViv	0.358064	0.000932
GO:0010827	regulation of glucose transport	pViv	0.15634	0.000705
GO:0016337	cell-cell adhesion	pViv	0.002316	0.000615
GO:0045725	positive regulation of glycogen biosynthetic process	pViv	0.316458	0.000806
GO:0008037	cell recognition	pViv	0.041274	0.000425
GO:0010907	positive regulation of glucose metabolic process	pViv	0.486254	0.000312
GO:0045913	positive regulation of carbohydrate metabolic process	pViv	0.561654	0.000731
GO:0010676	positive regulation of cellular carbohydrate metabolic process	pViv	0.561654	0.000731
GO:0030036	actin cytoskeleton organization	pViv	0.133792	8.55E-005
GO:0030029	actin filament-based process	pViv	0.099308	2.74E-005

### Case study 2: Reinterpretation of prior datasets

**Figure 10:**
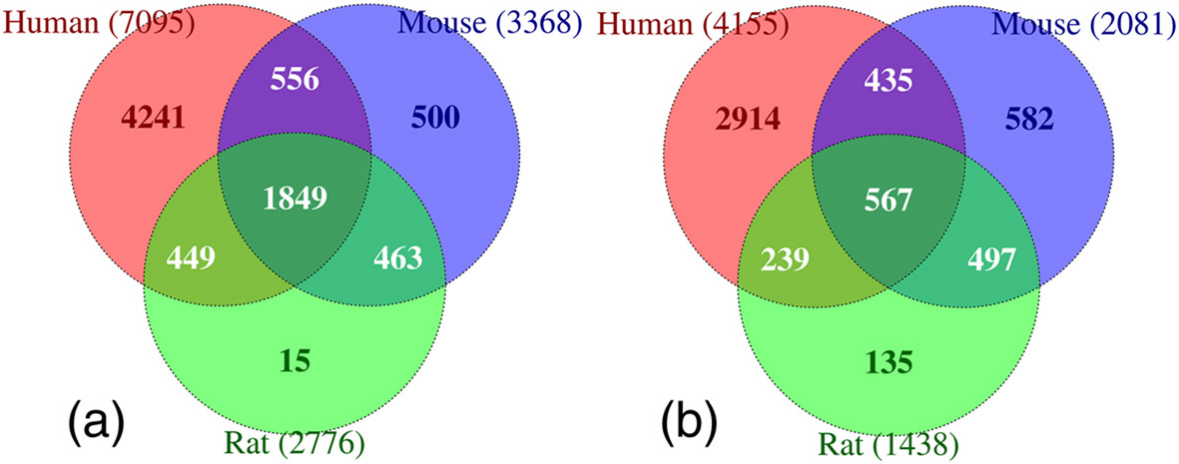
**Case study 2 - Reinterpretation of prior datasets using AbsIDconvert. (a)** Number of Incyte IDs (from a total of 8,392) mapping to the human, mouse and rat genomes within 5% of the maximum alignment score. **(b)** Incyte IDs with at least one Entrez ID found using AbsIDconvert.

Annotations used for DNA microarray studies quickly become out–of–date as more knowledge emerges about a species’ transcriptome. In addition, there are instances where one microarray platform may be used to measure gene products from a comparative species. For example, Incyte arrays spotted with human ESTs have been used to query gene expression levels in mouse and/or rat, based on the assumption that the human ESTs would bind to and provide measurements of the corresponding gene in rodents [69-71]. Using the original EST sequences spotted on the array from these studies, we sought to verify the current annotations of the ESTs, and also determine which rodent genes should bind the ESTs based on sequence alignment to the human, mouse, and rat genomes. Original EST sequences were found by searching two sources using the Incyte IDs supplied on the chip. The first source was the NCBI EST database, using a search string composed of “IMAGE:” and the Incyte clone ID number (identifies clones generated from the IMAGE consortium sequencing project). The second source was the Open Biosystems database (http://www.openbiosystems.com/), using a search string composed of “LIFESEQ” and the clone ID number. In some instances, multiple EST sequences were returned for each clone ID. A total of 8,392 sequences were downloaded and aligned to the genomes of human, rat, and mouse using AbsIDconvert with the default BLAT settings. The genome wide best alignment was found for each probe by considering only those alignments falling within 5% of the maximal alignment score (Figure 10(a)). Corresponding to each of these aligned coordinates, overlapping Entrez IDs were found for all three organisms. Out of the 7,095 human Incyte IDs which had corresponding genomic interval(s), 4,155 have at least one human Entrez ID associated with them. This number was 2,081 (out of 3,368) for mouse and 1,438 (out of 2,776) for rat (Figure 10(b)).

Homologous genes can be compared across species using NCBI’s Homologene resource [72] when gene names are known. However, if sequence information is available, it would be best to use that sequence information to determine if homology exists based on sequence conservation, particularly in cases where probes of known sequence are being used to measure a specific gene, such as in DNA microarrays or in–situ hybridization. Both methodologies were applied to the Incyte array used in [69-71].

For the Homologene based comparison, all of the Incyte IDs that map to at least one Entrez ID using AbsIDconvert were used to determine if a homologous gene exists, and if so, if there are corresponding entries for each of the species studied. Similarly, for those Incyte probes matching at least one Entrez ID, the sequence was used as a query into each of the other species using AbsIDconvert to determine if the probe maps to and overlaps an Entrez ID in a cross-species sense. As Table 7 indicates, using the Homologene conversion alone yields a high number of homologs (82% – 88%); however, using the sequence level information, it can be seen that a much lower percentage of probes (19% – 74%) actually map to known Entrez gene regions in the other species. These demonstrate that only a small number of the probes on the array should be utilized for cross species comparisons.

**Table 7:** Comparison of Homologene and sequence based homologs

**Organism**	**mapped †**	**Entrez ‡**	**Homol Â§**	**Human (Hom)**	**Mouse (Hom)**	**Rat (Hom)**	**Human (Seq)**	**Mouse (Seq)**	**Rat (Seq)**
Human	7095	4155	3854	–	3648 (88%)	3401 (82%)	–	1002 (24%)	806 (19%)
Mouse	3368	2081	1872	1794 (86%)	–	1715 (82%)	1002 (48%)	–	1064 (51%)
Rat	2776	1438	1263	1210 (84%)	1222 (85%)	–	806 (56%)	1064 (74%)	–

^a^mapped†:Number of probes mapped to Genome; Entrez‡:Mapped probes with Entrez ID; Homol*Â§*: Probes with Entrez ID as well as Homologene ID; Hom: Homologene Based Homologs; Seq: Sequence Based Homologs determined using AbsIDconvert.

### Case study 3: Meta–analytic studies across platforms

Meta-analysis enables the integration of many different experiments with a common research hypothesis. However, high-throughput -omics meta-analyses are hindered due to the heterogeneity of DNA microarray array designs (length and location of probes), data acquisition, analysis, and inter- and intra-study variability. Therefore, many meta-analyses use the same species or even the same array platform to mitigate some of these heterogeneities. However, many studies do still attempt to perform cross-platform and inter-species meta-analyses, and tools such as AILUN (Array Information Library Universal Navigator) [73], A-MADMAN (Annotation-based microarray data meta-analysis tool) [74], and LOLA (List Of Lists Annotated) [75] enable cross-species meta-analysis using Entrez ID, gene symbol or other IDs as a conversion intermediary. AbsIDconvert can perform cross-platform/-species analysis efficiently using the sequence based approach. We previously demonstrated that AbsIDconvert efficiently and accurately converted Affymetrix^®;^ HG_U133Plus2.0 probes into Agilent Cgh105a probes, among other types of conversions.

**Figure 11:**
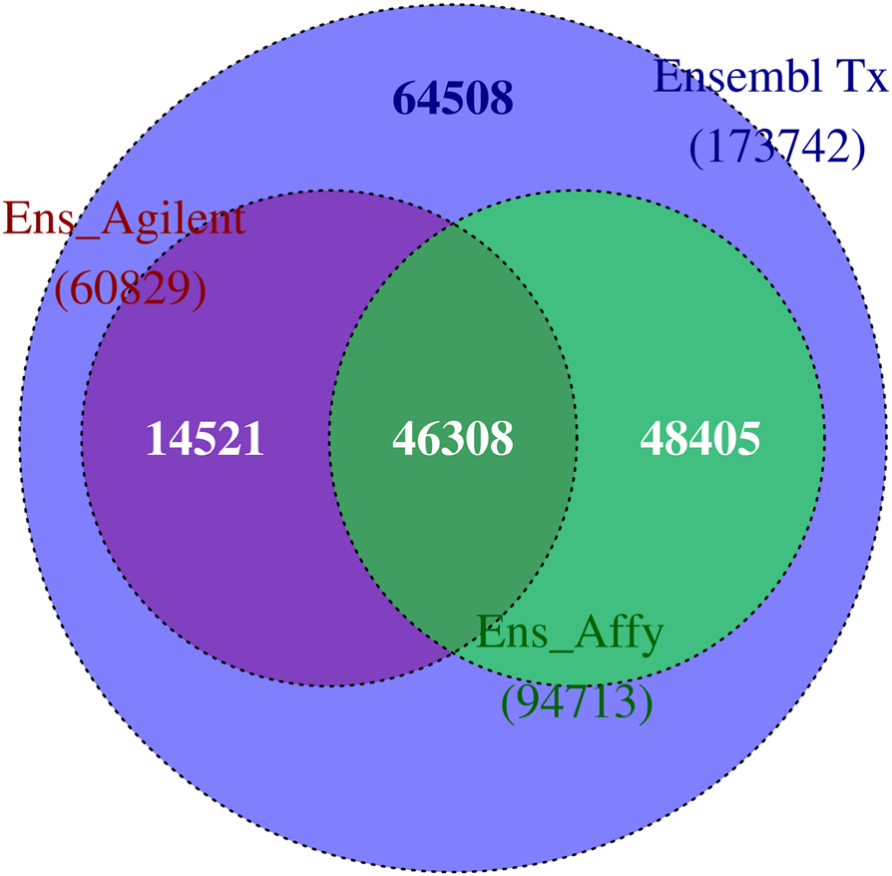
**Case study 3 - Cross-platform meta-analysis study using AbsIDconvert.** Ensembl transcripts mapped by Agilent Cgh 105a (purple) and Affymetrix^®;^ HG_U133Plus2.0 (green) probes.

To determine how comparable two microarray studies using different array platforms on a common organism could be, Affymetrix^®;^ HG_U133Plus2.0 and Agilent Cgh105a probe sequences were mapped and converted to corresponding human Ensembl transcripts using the default AbsIDconvert parameters. For the Affymetrix platform, 423,815 out of 603,158 probes were mapped to one or more transcripts, with 94,713 of the total Ensembl transcripts (173,742) being mapped (Figure 11). This leaves 79,029 Ensembl transcripts that were not mapped by any Affymetrix^®;^ probes. For Agilent, 27,184 (out of 99,026) mapped to 60,829 Ensembl transcripts. 79,029 (45% of the total) Ensembl transcripts do not have any mapped Agilent Cgh105a probes. The number of shared Ensembl transcripts between platforms was surprisingly small (46,308), indicating that each platform appears to have probe specific subsets of Ensembl transcripts. The number of Ensembl transcripts not probed by either platform was surprisingly large. This appears to be due to a lack of probes designed to bind those Ensembl transcripts, as the majority of unmapped transcripts are much shorter than those that are mapped (Additional file 8). As Figure 11 illustrates, 46,308 transcripts should be directly comparable between Affymetrix^®;^ HG_U133Plus2.0 and Agilent Cgh105a, while a large number of transcripts are not available in one or the other (or both) platforms.

## Conclusion

AbsIDconvert is the only known gene ID conversion tool based on genomic coordinates/intervals of which we are aware. This is a novel and important contribution in the realm of gene ID conversion due to the large variety of genetic entities in current use by biologists, the need to convert between them, and the fact that most biological entities (nucleic acid, protein entities etc.) have an associated sequence. Mapping of the entity sequence to a reference genome sequence provides the concomitant genomic interval that allows determination of other entities that have overlapping genomic intervals.

The interval basis of AbsIDconvert provides ease of flexibility with respect to any additions, deletions or updates of the underlying objects, requiring only adding of intervals, removing intervals, or modifying the intervals themselves, respectively. This makes it possible to easily keep the structure updated as the current state of biological knowledge changes. A major update is only required when the underlying genome changes, a fairly rare occurrence for most organisms, especially when compared to how often other genomic databases are modified.

These intervals also allow easy discovery of genetic entities that only partially overlap with queried IDs/intervals, or that are within a specified distance nearby. More frequently, researchers are interested in those genes that are near specific genomic intervals corresponding to various types of genetic control elements such as transcription factor binding sites, enhancers, untranslated regions, and hyper/hypo methylated regions. AbsIDconvert makes it easy to find those entities that overlap or lie nearby regions of interest. With the incorporation of a sequence mapping algorithm, AbsIDconvert integrates the determination of genomic intervals for any supplied sequence, making it possible to easily find and convert between IDs from any platform and organism, such as the examination of correspondence of the human EST clones with rat and mouse genes (case study 2) and of plasmodium and human genes (case study 1). We do not know of any other system that can easily accomplish these types of analyses.

AbsIDconvert can greatly facilitate the work of those who are involved in meta analyses studies. When comparing studies where either the species and / or platform varies, this methodology will have clear advantages over others as it is based on common genomic coordinates.

The use of an interval tree structure makes it possible to perform large conversions quickly and efficiently. This method is efficient while dealing with genomic intervals and has a significant advantage over other methods such as relational databases. Although theoretically limited by working memory, none of the interval trees generated and used by AbsIDconvert require more than 300MB of RAM on the deployed server, with the majority being rather small in size (less than 10 MB). If the data cannot fit into main memory, a method such as that proposed by Arge et al. [76,77] can be used that maintains the interval tree in secondary memory efficiently.

AbsIDconvert is provided as a web page at http://bioinformatics.louisville.edu/abid/, and is also available as a virtual machine for those wishing to run a local instance. Future work will include providing command line access, a RESTful interface, and modifying the interface to utilize a workflow management tool for genomic data such as GALAXY, where the primary data units are genomic sequences and intervals.

## Competing interests

The authors declare that they have no competing interests.

## Author’s contributions

FM was involved in all aspects of the project and was the main code developer. ECR and JCP designed the overall project goals. ECR was responsible for directing the project to completion. RMF played a large role in the project design, identifying sources of genetic entities for inclusion, and the design of the case studies. BJH provided critical assessment and usability design. All authors contributed to the preparation of the manuscript. All authors read and approved the final manuscript.

## Supplementary Material

Additional file 1 Table containing the NCBI Entrez gene symbol and the AbsIDconvert detected gene symbols for 83 Entrez IDs uniquely converted by AbsIDconvert. Click here for file

Additional file 2 Table containing information on the Entrez ID, gene symbol, gene type, and NCBI annotation for the 94 Entrez IDs converted by one or more conversion tools missed by AbsIDconvert. Click here for file

Additional file 3 Table containing information on the Entrez ID, RefSeq ID, and conversion results for Entrez IDs correctly converted to RefSeq IDs by MADGene that are missed by AbsIDconvert. Click here for file

Additional file 4 Table containing information on the chromosomal positions found for the five Entrez IDs that AbsIDconvert is unable to successfully convert to RefSeq IDs. Click here for file

Additional file 5 Table containing information on the Entrez IDs converted to RefSeq IDs by DAVID that do not have NCBI annotated RefSeq entries. Click here for file

Additional file 6 **Table containing information on the 38 Entrez IDs converted exclusively by AbsIDconvert to RefSeq IDs.** Thirty-five of the Entrez IDs are in agreement with NCBI’s Entrez annotation. Click here for file

Additional file 7 Table containing interval and overlapping RefSeq information for the five Entrez IDs converted exclusively by AbsIDconvert to RefSeq IDs that are not annotated in NCBI Entrez. Click here for file

Additional file 8 Figure showing the distribution of Ensembl transcript lengths for those transcripts either mapped or unmapped by either/both Affymetrix^®;^HG_U133Plus2.0 and Agilent Cgh105a microarray probes. Click here for file
