# Supplementary material for: AbsIDconvert: An absolute approach for converting genetic identifiers at different granularities
Source: BMC Bioinformatics. 2012 Sep 12;13:229. doi: 10.1186/1471-2105-13-229 (PMC3554462; doi:10.1186/1471-2105-13-229)
Supplement: Additional file 1 — Table containing the NCBI Entrez gene symbol and the AbsIDconvert detected gene symbols for 83 Entrez IDs uniquely converted by AbsIDconvert. [file 1471-2105-13-229-S1.pdf]

Table S1: Entrez IDs converted to gene symbols uniquely by AbsIDconvert.

EntrezID	Gene symbol (NCBI)	Gene type (NCBI)	Gene symbol (AbsIDconvert)
100505607	LOC100505607	miscRNA	LOC100505607
100505645	LOC100505645	miscRNA	LOC100505645
100505920	LOC100505920	miscRNA	LOC100505920
100505938	LOC100505938	miscRNA	LOC100505938
100505950	LOC100505950	miscRNA	LOC100505950
100506044	LOC100506044	miscRNA	LOC100506044
100506122	LOC100506122	miscRNA	LOC100506122
100506123	LOC100506123	miscRNA	LOC100506123
100506130	LOC100506130	miscRNA	LOC100506130
100506158	LOC100506158	miscRNA	LOC100506158
100506192	LOC100506192	miscRNA	LOC100506192
100506272	LOC100506272	miscRNA	LOC100506272
100506329	LOC100506329	miscRNA	LOC100506329
100506351	LOC100506351	miscRNA	LOC100506351
100506452	LOC100506452	miscRNA	LOC100506452
100506609	LOC100506609	miscRNA	LOC100506609
100506695	LOC100506695	miscRNA	PHF21B, LOC100506695
100506837	LOC100506837	miscRNA	LOC100506837
100507153	LOC100507153	miscRNA	LOC100507153
100507205	LOC100507205	miscRNA	LOC100507205
100507389	LOC100507389	miscRNA	LOC100507389
100507581	LOC100507581	miscRNA	LOC100507581
100507615	LOC100507615	miscRNA	LOC100507615
100507672	LOC100507672	miscRNA	PPARD, LOC100507672
100529145	TEN1-CDK3	miscRNA	C17ORF106-CDK3, TEN1, CDK3
100529211	C17orf61-PLSCR3	miscRNA	C17ORF61-PLSCR3, PLSCR3, C17ORF61
100630923	LOC100630923	miscRNA	LOC100630923, LOC100289561, PRKRIP1
100652780	LOC100652780	other	LOC100652780
100329135	LOC100329135	protein-coding	LOC100329135
100505549	LOC100505549	protein-coding	LOC100505549
100507421	LOC100507421	protein-coding	LOC100507421, LOC100130169
100652826	LOC100652826	protein-coding	LOC100652826
100303743	LOC100303743	pseudo	WWOX, LOC100303743
100418753	LOC100418753	pseudo	LOC100418753
100418754	LOC100418754	pseudo	LOC100418754
100418951	LOC100418951	pseudo	LOC100418951
100418955	LOC100418955	pseudo	LOC100418955
100419014	LOC100419014	pseudo	LOC100419014
100419017	LOC100419017	pseudo	LOC100419017
100419108	LOC100419108	pseudo	RBMS1, LOC100419108
100419553	LOC100419553	pseudo	LOC100419553
100419621	LOC100419621	pseudo	TADA2A, LOC100419621
100419694	LOC100419694	pseudo	LOC100419694
100419779	LOC100419779	pseudo	LOC100419779
100419814	LOC100419814	pseudo	CUL3, LOC100419814

Continued on next page...

Table S1 – continued from previous page

Entrez ID	Gene symbol (NCBI)	Gene type (NCBI)	Gene symbol (AbsIDconvert)
100419892	LOC100419892	pseudo	LOC100419892
100419951	LOC100419951	pseudo	LOC100419951
100419986	LOC100419986	pseudo	LOC100419986
100420064	LOC100420064	pseudo	LOC100420064
100420177	LOC100420177	pseudo	LOC100420177
100420305	LOC100420305	pseudo	LOC100420305
100420358	LOC100420358	pseudo	LOC100420358
100420741	LOC100420741	pseudo	LOC100420741
100420886	LOC100420886	pseudo	WDR35, LOC100420886
100420949	LOC100420949	pseudo	LOC100287225, LOC100420949
100421028	LOC100421028	pseudo	LOC100421028
100421121	LOC100421121	pseudo	LOC100421121
100421437	LOC100421437	pseudo	ZNF148, LOC100421437
100421471	LOC100421471	pseudo	LOC100421471
100421494	LOC100421494	pseudo	LOC100421494
100421695	LOC100421695	pseudo	LOC100421695
100422265	LOC100422265	pseudo	LOC100422265
100422284	LOC100422284	pseudo	LOC100422284
100422299	LOC100422299	pseudo	LOC100422299
100422510	LOC100422510	pseudo	LOC100422510
100422524	LOC100422524	pseudo	MLLT10, LOC100422524
100422671	LOC100422671	pseudo	LOC100422671
100500934	LOC100500934	pseudo	LOC100500934
100507388	LOC100507388	pseudo	LOC100505505, LOC100507388
100507595	LOC100507595	pseudo	LRRC33, LOC100507595
100526736	LOC100526736	pseudo	LOC100526736
100533622	LOC100533622	pseudo	LOC100533622
100533658	LOC100533658	pseudo	LOC100533658
100533661	LOC100533661	pseudo	LOC100533661
100533663	LOC100533663	pseudo	LOC100533663
100533732	LOC100533732	pseudo	LOC100533732
100533846	LOC100533846	pseudo	LOC100533846
100631258	LOC100631258	pseudo	NELL1, LOC100631258
100652752	LOC100652752	pseudo	LOC100652752
100652792	LOC100652792	pseudo	LOC100652792
100505530	FLJ45825	unknown	LOC100505530
100506282	LOC100506282	unknown	LOC100506282
100506342	LOC100506342	unknown	LOC100506342
