# Supplementary material for: AbsIDconvert: An absolute approach for converting genetic identifiers at different granularities
Source: BMC Bioinformatics. 2012 Sep 12;13:229. doi: 10.1186/1471-2105-13-229 (PMC3554462; doi:10.1186/1471-2105-13-229)
Supplement: Additional file 2 — Table containing information on the Entrez ID, gene symbol, gene type, and NCBI annotation for the 94 Entrez IDs converted by one or more conversion tools missed by AbsIDconvert. [file 1471-2105-13-229-S2.pdf]

Table S2: Entrez IDs converted to gene symbol by HMS & ID, DAVID and MADGene missed by AbsIDConvert.

EntrezID	Gene symbol (NCBI)	Gene type (NCBI)	NCBI annotation	HMS & ID	DAVID	MADGene
286750	–	–	replaced with Gene ID: 9414	–	DFNA51	DFNA51
5374	–	–	replaced with Gene ID: 390831	–	PMM2P1	PMM2P1
728795	–	–	replaced with Gene ID: 100420746	–	LOC728795	hCG_1644355
493620	–	–	replaced with Gene ID: 100418887	–	TAGLN2P1	TAGLN2P1
645128	–	–	replaced with Gene ID: 100288486	–	LOC645128	LOC645128
100507343	CPB2-AS1	miscRNA	Not on current assembly	CPB2-AS1	–	–
100129836	COL4A2-AS2	miscRNA	Not on current assembly	COL4A2-AS2	LOC100129836	LOC100129836
57234	FAM91A2	miscRNA	Not on current assembly	FAM91A2	FAM91A2	FAM91A2
693204	MIR619	miscRNA	Not on current assembly	MIR619	MIR619	MIR619
729634	KRT18P26	pseudo	Not on current assembly	KRT18P26	KRT18P19, KRT18, KRT18P26	KRT18P26
100189058	TRNAQ9	tRNA	Not on current assembly	TRNAQ9	TRNAQ9	TRNAQ9
283486	LINC00567	unknown	Not on current assembly	LINC00567	LOC283486	LOC283486
100131497	LOC100131497	miscRNA	Not on current assembly	–	LOC100131497	LOC100131497
401021	LOC401021	miscRNA	Not on current assembly	–	LOC401021	LOC401021
729683	LOC729683	miscRNA	Not on current assembly	–	LOC729683	LOC729683
100288085	DYZ1L5	other	Not on current assembly	–	LOC100288085	LOC100288085
100131004	LOC100131004	protein-coding	Not on current assembly	–	LOC100131004	LOC100131004
100131310	LOC100131310	protein-coding	Not on current assembly	–	LOC100131310	LOC100131310
100132365	LOC100132365	protein-coding	Not on current assembly	–	LOC100132365	LOC100132365
100286906	LOC100286906	protein-coding	Not on current assembly	–	LOC100286906	LOC100286906
100289058	LOC100289058	protein-coding	Not on current assembly	–	LOC100289058	LOC100289058

Continued on next page...

Table S2 – continued from previous page

Entrez ID	Gene symbol (NCBI)	Gene type (NCBI)	NCBI annotation	HMS & ID	DAVID	MADGene
653541	LOC653541	protein-coding	Not on current assembly	–	LOC399839, HPX-2, LOC728410, LOC653541, LOC653548, LOC653544, LOC653543, LOC653545, LOC440014, LOC440013, LOC441056, LOC728022, DUX4, LOC652119, LOC440017	LOC653541
727961	LOC727961	protein-coding	Not on current assembly	–	LOC727961	hCG_1776047
100128019	LOC100128019	unknown	Not on current assembly	–	LOC100128019	LOC100128019
100129894	LOC100129894	unknown	Not on current assembly	–	LOC100129894	LOC100129894
100131043	LOC100131043	unknown	Not on current assembly	–	LOC100131043	LOC100131043
100288869	LOC100288869	unknown	Not on current assembly	–	LOC100288869	LOC100288869
400943	LOC400943	unknown	Not on current assembly	–	UNQ5830	UNQ5830
440479	FLJ34223	unknown	Not on current assembly	–	FLJ34223	FLJ34223
645895	LOC645895	unknown	Not on current assembly	–	LOC645895	LOC645895
645967	LOC645967	unknown	Not on current assembly	–	LOC645967	LOC645967
648149	LOC648149	unknown	Not on current assembly	–	LOC648149	LOC648149
727799	LOC727799	unknown	Not on current assembly	–	LOC727799	LOC727799
693128	MIR548B	miscRNA	Not annot. on reference assembly	MIR548B	MIR548B	MIR548B
28332	IGHD2OR15-2B	other	Not annot. on reference assembly	IGHD2OR15-2B	IGHD2OR15-2B	IGHD2OR15-2B
3506	IGHJ@	other	Not annot. on reference assembly	IGHJ@	IGHJ@	IGHJ@
100313795	PIRC73	other	Not annot. on reference assembly	PIRC73	–	–
100313815	PIRC54	other	Not annot. on reference assembly	PIRC54	–	–
28301	IGHV3OR16-15	pseudo	Not annot. on reference assembly	IGHV3OR16-15	IGHV3OR16-15	IGHV3OR16-15
28854	IGKV3OR2-5	pseudo	Not annot. on reference assembly	IGKV3OR2-5	IGKV3OR2-5	IGKV3OR2-5

Continued on next page...

Table S2 – continued from previous page

Entrez ID	Gene symbol (NCBI)	Gene type (NCBI)	NCBI annotation	HMS & ID	DAVID	MADGene
28861	IGKV2OR2-1	pseudo	Not annot. on reference assembly	IGKV2OR2-1	IGKV2OR2-1	IGKV2OR2-1
4699	NDUFA5P1	pseudo	Not annot. on reference assembly	NDUFA5P1	NDUFA5P1	NDUFA5P1
654813	P2RY10P1	pseudo	Not annot. on reference assembly	P2RY10P1	P2RY10P1	P2RY10P1
100189517	TRNAN35	tRNA	Not annot. on reference assembly	TRNAN35	TRNAN35P	TRNAN35P
25784	DGCR12	unknown	Not annot. on reference assembly	DGCR12	DGCR12	DGCR12
3405	IDDM6	unknown	Not annot. on reference assembly	IDDM6	IDDM6	IDDM6
4375	MRX11	unknown	Not annot. on reference assembly	MRX11	MRX11	MRX11
554188	FCMTE2	unknown	Not annot. on reference assembly	FCMTE2	FCMTE2	FCMTE2
594832	MYP11	unknown	Not annot. on reference assembly	MYP11	MYP11	MYP11
6893	TAPVR1	unknown	Not annot. on reference assembly	TAPVR1	TAPVR1	TAPVR1
7889	PSORS3	unknown	Not annot. on reference assembly	PSORS3	PSORS3	PSORS3
882	CCAL1	unknown	Not annot. on reference assembly	CCAL1	CCAL1	CCAL1
89760	MRX75	unknown	Not annot. on reference assembly	MRX75	MRX75	MRX75
100302562	MENAQ2	unknown	Not annot. on reference assembly	–	–	MENAQ2
400579	FLJ35934	miscRNA	Not annot. on reference assembly	–	FLJ35934	FLJ35934
286009	LOC286009	other	Not annot. on reference assembly	–	LOC286009	tcag7.929
387281	LCRB	other	Not annot. on reference assembly	–	LCRB	LCRB
402469	LOC402469	other	Not annot. on reference assembly	–	LOC402469	tcag7.1056
26101	DKFZP564M1462	protein-coding	Not annot. on reference assembly	–	DKFZP564M1462	DKFZP564M1462
283911	LOC283911	protein-coding	Not annot. on reference assembly	–	LOC283911	LOC283911
55547	HAB1	protein-coding	Not annot. on reference assembly	–	HAB1	HAB1
653486	LOC653486	protein-coding	Not annot. on reference assembly	–	LOC653486	hCG_1741344
100133452	LOC100133452	pseudo	Not annot. on reference assembly	–	LOC100133452	LOC100133452
100292981	LOC100292981	pseudo	Not annot. on reference assembly	–	LOC100292981	LOC100292981
100294336	LOC100294336	pseudo	Not annot. on reference assembly	–	LOC100294336	LOC100294336
647349	LOC647349	pseudo	Not annot. on reference assembly	–	LOC647349	LOC647349
652073	LOC652073	pseudo	Not annot. on reference assembly	-	LOC652073	LOC652073
730535	LOC730535	pseudo	Not annot. on reference assembly	–	LOC730535	LOC730535
100049541	STUT1	unknown	Not annot. on reference assembly	–	STUT1	STUT1
100134822	LOC100134822	unknown	Not annot. on reference assembly	–	LOC100134822	LOC100134822

Continued on next page...

Table S2 – continued from previous page

Entrez ID	Gene symbol (NCBI)	Gene type (NCBI)	NCBI annotation	HMS & ID	DAVID	MADGene
100188748	STHAG5	unknown	Not annot. on reference assembly	–	STHAG5	STHAG5
100188844	MAFD6	unknown	Not annot. on reference assembly	–	MAFD6	MAFD6
100188852	SHEP8	unknown	Not annot. on reference assembly	–	SHEP8	SHEP8
100188853	BMND7	unknown	Not annot. on reference assembly	–	BMND7	BMND7
100190985	IS5	unknown	Not annot. on reference assembly	–	IS5	IS5
100271922	BFIC4	unknown	Not annot. on reference assembly	–	BFIC4	BFIC4
100293044	LOC100293044	unknown	Not annot. on reference assembly	–	LOC100293044	LOC100293044
338030	GLM1	unknown	Not annot. on reference assembly	–	GLM1	GLM1
414058	ACRPV	unknown	Not annot. on reference assembly	–	ACRPV	ACRPV
474225	RA5	unknown	Not annot. on reference assembly	–	RA5	RA5
474261	BP15	unknown	Not annot. on reference assembly	–	BP15	BP15
474285	OA1	unknown	Not annot. on reference assembly	–	OA1	GPR143
474294	BW19	unknown	Not annot. on reference assembly	–	BW19	BW19
474295	BW20	unknown	Not annot. on reference assembly	–	BW20	BW20
50989	HMSNO	unknown	Not annot. on reference assembly	–	HMSNO	HMSNO
544599	AASTH45	unknown	Not annot. on reference assembly	–	AASTH45	AASTH45
544616	COHEN2	unknown	Not annot. on reference assembly	–	COHEN2	COHEN2
780911	TQDS	unknown	Not annot. on reference assembly	–	TQDS	TQDS
780925	CHMRQ	unknown	Not annot. on reference assembly	–	CHMRQ	CHMRQ
7834	PCAP	unknown	Not annot. on reference assembly	–	PCAP	PCAP
8008	BDMF	unknown	Not annot. on reference assembly	–	BDMF	BDMF
8041	TP250	unknown	Not annot. on reference assembly	–	TP250	TP250
8173	CNSN	unknown	Not annot. on reference assembly	–	CNSN	CNSN
8205	TAM	unknown	Not annot. on reference assembly	–	TAM	TAM
