# Supplementary material for: AbsIDconvert: An absolute approach for converting genetic identifiers at different granularities
Source: BMC Bioinformatics. 2012 Sep 12;13:229. doi: 10.1186/1471-2105-13-229 (PMC3554462; doi:10.1186/1471-2105-13-229)
Supplement: Additional file 3 — Table containing information on the Entrez ID, RefSeq ID, and conversion results for Entrez IDs correctly converted to RefSeq IDs by MADGene that are missed by AbsIDconvert. [file 1471-2105-13-229-S3.pdf]

Table S3: Entrez IDs converted to Refseq by MADGene missed by AbsIDConvert.

EntrezID	RefSeq (NCBI)	MADGene	DAVID	Onto-Translate
6080	NR_002907	NR_002907	NR_004385, NR_004406, NR_004404, NR_002907, NR_004386	NR_002907
26822	NR_000022	NR_000022	NR_001452, NR_001454, NR_001453, NR_003125, NR_000022	NR_000022
100302146	NR_031634	NR_031634	-	-
100302193	NR_031656	NR_031656	-	-
100302167	NR_031629	NR_031629	NR_031629	-
