# Supplementary material for: AbsIDconvert: An absolute approach for converting genetic identifiers at different granularities
Source: BMC Bioinformatics. 2012 Sep 12;13:229. doi: 10.1186/1471-2105-13-229 (PMC3554462; doi:10.1186/1471-2105-13-229)
Supplement: Additional file 4 — Table containing information on the chromosomal positions found for the five Entrez IDs that AbsIDconvert is unable to successfully convert to RefSeq IDs. [file 1471-2105-13-229-S4.pdf]

Table S4: Genomic intervals found by AbsIDconvert for the five unmapped Entrez IDs found by MADGene.

Entrez ID	chromosome	start	end	width	strand
6080	chr1	28833877	28834083	207	+
26822	chr11	17096200	17096291	92	-
100302146	chr20	49231173	49231322	150	-
100302193	chr4	102251459	102251571	113	-
100302167	chr9	69002239	69002321	83	-
