# Supplementary material for: AbsIDconvert: An absolute approach for converting genetic identifiers at different granularities
Source: BMC Bioinformatics. 2012 Sep 12;13:229. doi: 10.1186/1471-2105-13-229 (PMC3554462; doi:10.1186/1471-2105-13-229)
Supplement: Additional file 5 — Table containing information on the Entrez IDs converted to RefSeq IDs by DAVID that do not have NCBI annotated RefSeq entries. [file 1471-2105-13-229-S5.pdf]

Table S5: Entrez IDs to RefSeq conversion by DAVID, with missing annotation from NCBI.

EntrezID	DAVID	NCBI Entrez annotation for DAVID RefSeq
100129552	NM_001029	6231
285176	NM_006013, NR_026898	6134
388474	NM_000972	6130
440991	NM_001005	6188
642538	NM_006333, NM_173177	10438
642585	NM_003374	7416
644634	NR_027002	388692
646050	NM_022831	64853
653252	NM_006327	100287932
727828	NM_001164397	642446
727984	NM_001035006, NM_000985	6139, 6140
728513	NM_032882	84968
728533	NM_014761	9798
728698	NM_001416, NR_002912	1973
728953	NM_001022	6223
728970	NM_025113	80183
729163	NM_001444	2171
729458	NM_144614	125997
729992	NM_003932	6767
81458	NM_001001824	403239
