# Supplementary material for: AbsIDconvert: An absolute approach for converting genetic identifiers at different granularities
Source: BMC Bioinformatics. 2012 Sep 12;13:229. doi: 10.1186/1471-2105-13-229 (PMC3554462; doi:10.1186/1471-2105-13-229)
Supplement: Additional file 6 — Table containing information on the 38 Entrez IDs converted exclusively by AbsIDconvert to RefSeq IDs. Thirty-five of the Entrez IDs are in agreement with NCBI’s Entrez annotation. [file 1471-2105-13-229-S6.pdf]

Table S6: Entrez IDs converted to RefSeq IDs exclusively by AbsIDconvert.

EntrezID	RefSeq	AbsIDconvert
81104	-	NR_015416
100505905	-	NM_001256876, NM_001256877
400433	-	NR_033787
100131381	-	NR_029697
100652874	-	NR_046251, NR_046252, NR_046253, NR_046254, NR_046255
100463488	NM_001190708	NM_001190708
100271846	NM_001191055	NM_001191055
642612	NM_001195234	NM_001195234
100507421	NM_001195278	NM_001195278
100287466	NM_001242319	NM_001242319, NM_032882
100313837	NR_031576	NR_031576
100329109	NR_033248	NR_033248
647135	NR_034178	NR_034178
100422851	NR_036200	NR_036200
100422860	NR_036251	NR_036251
284648	NR_036490	NR_036490
100289373	NR_036531, NR_036532	NR_036531, NR_036532
100500878	NR_037450	NR_037450
100506548	NR_037665	NR_037665
100359394	NR_037842	NR_037842
100507582	NR_037903	NR_037903
100505687	NR_038301, NR_038302	NR_038301, NR_038302
554206	NR_038379	NR_038379
729444	NR_038388	NR_038388
100129464	NR_038428	NR_038428
253962	NR_038439	NR_038439
147093	NR_038442	NR_038442
284865	NR_038460	NR_038460
100507401	NR_038909	NR_038909
100506241	NR_038954	NR_038954
100616164	NR_039616	NR_039616
100616469	NR_039627	NR_039627
100616499	NR_039634	NR_039634, NR_039636
100616399	NR_039635	NR_039635
100616315	NR_039942	NR_039942
284395	NR_040029	NR_040029
81343	NR_045005	NR_045005
440519	NR_045525	NR_045525
