# Supplementary material for: AbsIDconvert: An absolute approach for converting genetic identifiers at different granularities
Source: BMC Bioinformatics. 2012 Sep 12;13:229. doi: 10.1186/1471-2105-13-229 (PMC3554462; doi:10.1186/1471-2105-13-229)
Supplement: Additional file 7 — Table containing interval and overlapping RefSeq information for the five Entrez IDs converted exclusively by AbsIDconvert to RefSeq IDs that are not annotated in NCBI Entrez. [file 1471-2105-13-229-S7.pdf]

Table S7: Genomic intervals for AbsIDconvert Entrez to RefSeq conversion.

chromosome	start	end	width	strand	name	chromosome	start	end	width	strand	name
chr15	22332368	22333348	981	+	81104	chr15	22332387	22332775	389	+	NR_015416
chr9	540552	549535	8984	+	100505905	chr9	540507	540666	160	+	NM_001256876
chr9	540552	549535	8984	+	100505905	chr9	549106	549720	615	+	NM_001256877
chr15	85046982	85049663	2682	+	400433	chr15	85046594	85050248	3655	+	NR_033787
chr11	43600529	43606151	5623	+	100131381	chr11	43602943	43603032	90	+	NR_029697
chr3	14961857	14989931	28075	-	100652874	chr3	14984285	14987660	3376	-	NR_046252
chr3	14961857	14989931	28075	-	100652874	chr3	14984285	14987660	3376	-	NR_046253
chr3	14961857	14989931	28075	-	100652874	chr3	14984285	14987660	3376	-	NR_046254
chr3	14961857	14989931	28075	-	100652874	chr3	14984285	14987660	3376	-	NR_046255
chr3	14961857	14989931	28075	-	100652874	chr3	14984285	14987660	3376	-	NR_046251
chr3	14961857	14989931	28075	-	100652874	chr3	14988593	14989011	419	-	NR_046252
chr3	14961857	14989931	28075	-	100652874	chr3	14988616	14989011	396	-	NR_046253
chr3	14961857	14989931	28075	-	100652874	chr3	14988620	14989011	392	-	NR_046254
chr3	14961857	14989931	28075	-	100652874	chr3	14989245	14989399	155	-	NR_046255
chr3	14961857	14989931	28075	-	100652874	chr3	14989519	14989947	429	-	NR_046251
