# Supplementary figures and images for: AbsIDconvert: An absolute approach for converting genetic identifiers at different granularities
Source: BMC Bioinformatics. 2012 Sep 12;13:229. doi: 10.1186/1471-2105-13-229 (PMC3554462; doi:10.1186/1471-2105-13-229)

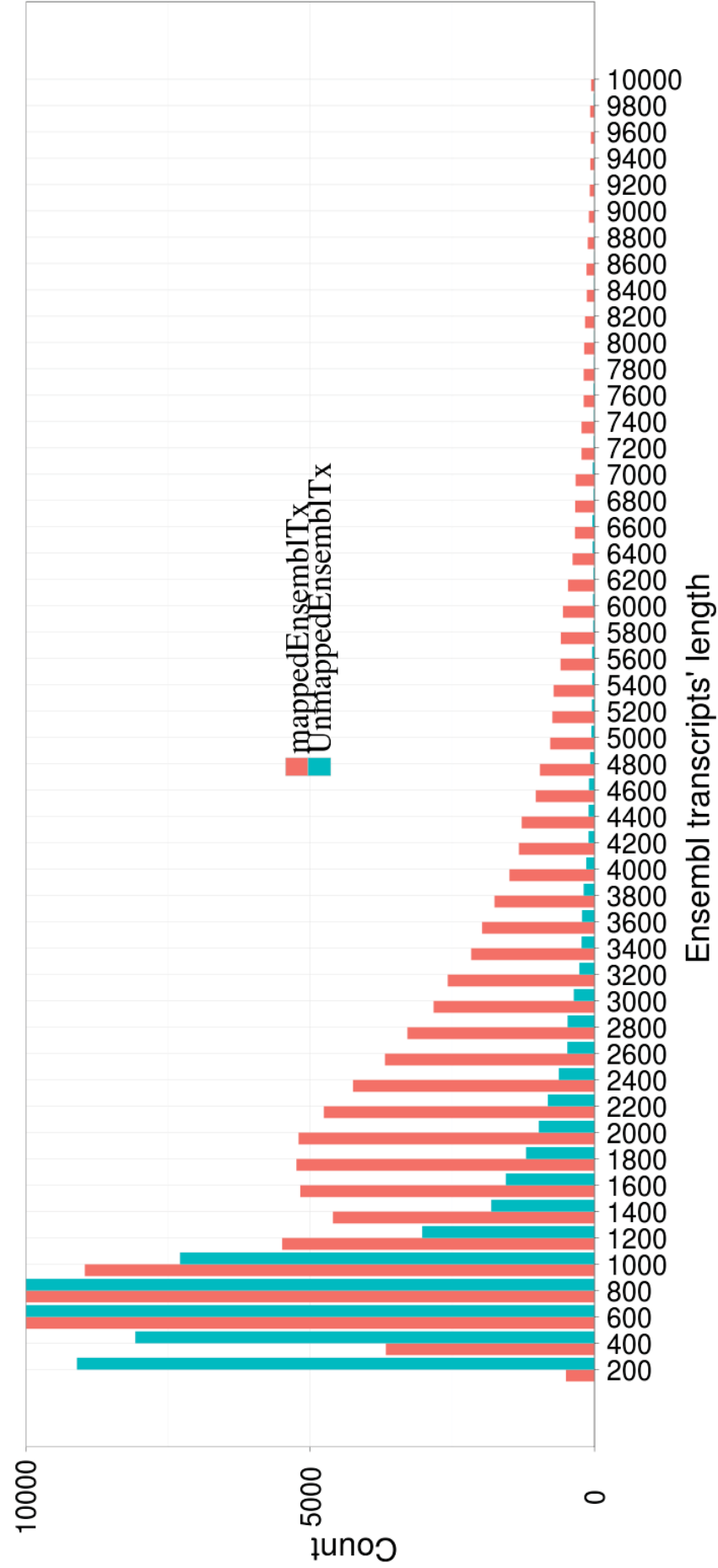

Figure S1: Case Study 3 - Ensembl transcript lengths mapped / unmapped by probes.

Supplement: Additional file 8 — Figure showing the distribution of Ensembl transcript lengths for those transcripts either mapped or unmapped by either/both Affymetrix®;HG_U133Plus2.0 and Agilent Cgh105a microarray probes. [file 1471-2105-13-229-S8.pdf]
